# Preparation of High-Purity Vanadium Metal by Iodide Refining Based on Numerical Simulation Using SIMPLE Algorithm

**DOI:** 10.3390/ma19194261

**Published:** 2026-10-08

**Authors:** Zhifang Hu, Bowen Gao, Honglin Jiang, Qidong Zhang, Yanxi Yin

**Affiliations:** 1 National Engineering Research Center for Environment-Friendly Metallurgy in Producing Premium Non-Ferrous Metals, Beijing 101407, China; 2 GRINM Resources and Environment Tech. Co., Ltd., Beijing 101407, China; 3 General Research Institute for Nonferrous Metal, Beijing 100088, China; 4 Beijing Engineering Research Center of Strategic Nonferrous Metals Green Manufacturing Technology, Beijing 101407, China

**Keywords:** high-purity vanadium, iodide refining, mass transport, flow transition, purification

## Abstract

In this study, the iodide refining of high-purity vanadium metal was investigated experimentally and by two-dimensional numerical simulations based on the SIMPLE (Semi-Implicit Method for Pressure-Linked Equations) algorithm. The effects of system temperature, K values (characterizing the deposition filament temperature), initial iodine amount, and raw material packing configuration on the deposition rate, crystal morphology, and mass transport were studied in a custom-built iodide apparatus. The experimental results showed that the deposition rate exhibited a bell-shaped dependence on the source temperature. The optimal source temperature and optimal K value were 1123–1173 K and 40–50, respectively, at which the average deposition rate reached its peak. Both the average deposition rate and the average reaction time increased slightly with increasing initial iodine amount, but the increases were limited. Full loading of the raw material yielded crystal rods of uniform size. Vanadium crystal rods with a diameter of approximately 20 mm were obtained after refining. The metallic impurities Fe, Cr, Al, and Si were reduced to 0.025–0.033 wt.%, <0.001 wt.%, <0.001 wt.%, and <0.002 wt.%, respectively, while the interstitial impurities O, C, and N were reduced to <0.01 wt.%, <0.002 wt.%, and <0.002 wt.%, respectively, indicating that iodide refining can effectively reduce the contents of both interstitial and metallic impurities in vanadium metal. Numerical simulation results showed that the mass transport rate decreased monotonically with increasing total pressure, increased with rising temperature owing to the enhanced VI_2_ partial-pressure difference, and decreased with increasing transport distance. The flow pattern transitioned from pure diffusion/advection at 300 Pa to transitional vortices at 1000 Pa, and finally to fully developed recirculation at 3000 Pa. The simulation results are qualitatively consistent with the experimental observations, providing a theoretical basis for process optimization. To further improve the purity and scale-up potential of the process, it is necessary to conduct more in-depth research, including the development of fully coupled models that incorporate gas-phase dissociation, non-equilibrium surface kinetics, and composition-dependent mixture properties, as well as three-dimensional simulations and reactor material optimization.

## 1. Introduction

High-purity metal vanadium (≥99.95% or more) is in critical demand across various sectors, including integrated circuits [1,2,3], nuclear industry [4,5,6,7], aerospace [8,9], and optoelectronic information [10,11,12], etc. Purity is fundamental to material design and performance [13], and even trace impurities, especially gaseous or interstitial impurities, including oxygen, nitrogen, and carbon, at the ppm or sub-ppm level can adversely affect the performance of materials and devices [14,15,16]. In addition, fundamental studies have revealed that trace impurities significantly influence the physical and chemical properties of high-purity metals, indicating that pure materials may serve as new functional materials, opening new frontiers in materials science and engineering [17].

The increasing demands for material specifications in high-performance information devices have led to a rapid growth in the need for ultra-high-purity metals [17,18]. A number of methods such as the active metal thermal reduction [19,20], electrorefining [21,22], electron-beam melting [23], etc., have been intensively investigated for obtaining of high-purity metal vanadium in recent decades. These refining methods exhibit unique advantages in their respective application scenarios. Due to the high chemical affinity of vanadium for interstitial impurities [17,24], it is particularly challenging to deeply remove these impurities. For example, the oxygen content can typically be reduced to about 200–300 ppm using the conventional methods above [21,22,23].

The iodide refining process is a very effective refining method for leaving behind interstitial impurities, such as oxygen, carbon, and nitrogen, which has been verified in the production of high-purity titanium, zirconium, and hafnium [25,26,27]. Also, Metallic impurities generally transport poorly, depending on the behavior of individual iodides. In the early 1960s, O. N. Carlson and C. V. Owen [28] produced a vanadium crystal bar using the iodization method under conditions where the feed and filament temperatures were 800 °C and 1300 °C, respectively. However, further research on this topic has rarely been reported since then, even though many related literature references mention this approach.

The mass transport of the gaseous mixture between the source and deposition zones plays a decisive role in determining the actual deposition rate and deposit quality on the filament during iodide refining; however, its underlying mechanisms and key influencing parameters remain unclear. The transport phenomena in the physical vapor transport (PVT) process within a closed system have been extensively studied through numerical simulation [29,30]. However, mass transport in PVT processes is not consistent with that in iodide refining. In PVT, the carrier gas is inert and is rejected by the source and crystal surfaces; unlike PVT, the iodine carrier gas in the iodide refining process is not inert but reacts with the source and deposition surfaces, so that the mass fluxes and mass concentrations on both surfaces differ significantly from those derived in PVT. Also, there are few modeling studies on CVT [31,32,33,34]. Zappola et al. [31] developed a two-dimensional rectangular model for the GeI_2_/GeI_4_ system with Boussinesq approximation and non-equilibrium surface kinetics-related boundary conditions, to simulate the flow pattern changes with temperature and local gravity. Ran Zuo et al. [33,34] simulated the mass transport process for the ZnS-I_2_ system. Nevertheless, these studies neglected compressibility and gas-phase reactions; simplified thermophysical parameters, are valid only for small temperature gradients and small variations in mean molecular weight. Moreover, because of the complexity of the surface-kinetics-related boundary conditions, those models are difficult to apply directly to the vanadium iodide refining system, although they can still offer qualitative reference for the present work.

In this study, experiments for preparation of high-purity vanadium metal by iodide refining were carried out in a custom-built apparatus, examining the effects of system temperature, K value (characterizing the deposition filament temperature), initial iodine amount, and raw material packing configuration. A two-dimensional numerical simulation based on the SIMPLE (Semi-Implicit Method for Pressure-Linked Equations) algorithm was also conducted to investigate the dependence of the mass transport rate in the closed iodide refining process on the system pressure, system temperature, and model geometry. The specific objectives are as follows: (i) to determine the optimal process parameters for high deposition rate and uniform crystal growth; (ii) to quantify the purification effect on metallic and interstitial impurities; and (iii) to qualitatively reveal the coupled diffusion–convection transport mechanism and flow-regime transitions. The present work is not only applicable to the iodide refining of vanadium but can also provide qualitative predictions for mass transport in the iodide refining processes of other metals.

## 2. Iodide Refining Mechanism

The fundamental principle of iodide refining for metals is to exploit the temperature-dependent reactions between iodine and the target metal, with iodine serving as the transport medium to selectively carry metal atoms from a low-temperature source zone to a high-temperature deposition zone for nucleation and growth. This cyclic transport effectively purifies the metal, and iodine acts as a “sorter and carrier” [26,27].

**Figure 1:**
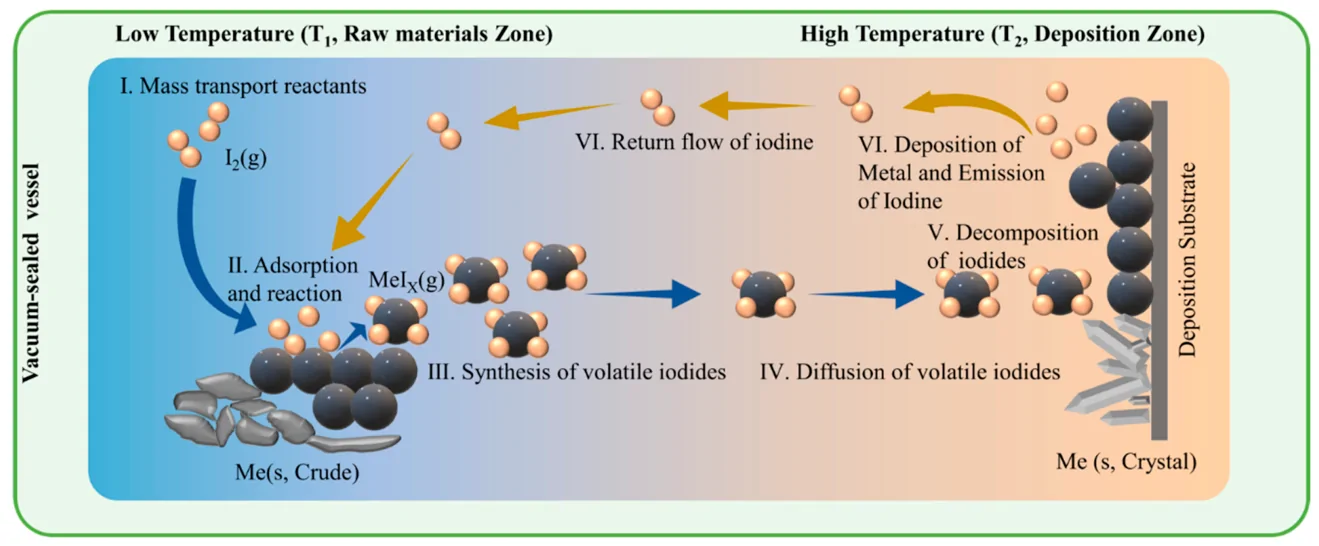
Scheme of iodide refining process, adapted from Refs. [26,27] and references therein.

As illustrated in Figure 1, the general principle of transport reactions can be seen clearly: the raw material is transformed reversibly into gaseous products by means of the transport agent (I_2_). At another temperature, and therefore a changed equilibrium position, the back reaction sets in, which results in the deposition of the solid from the gas phase in the crystallization zone. It is common to characterize the source and deposition temperatures with T_1_ and T_2_, respectively, with T_1_ representing the lower temperature. Therefore, exothermic reactions always transport from T_1_ to T_2_; conversely, endothermic reactions from T_2_ to T_1_. Such a vapor transport reaction can be described by Equation (1):

(1) $$Me\left(s\right)+\frac{x}{2}I_{2}\left(g\right)\overset{T_{1}}{\underset{T_{2}}{⇌}}MeI_{x}(g)$$

Here, the complete transport process can be separated into three stages: (1) heterogeneous reaction between gaseous I_2_ and solid Me in the raw-material zone; (2) transport of gaseous species (I_2_ and MeI_x_) through the chamber; and (3) heterogeneous reaction to re-form solid Me on the substrate surface. Under a low total pressure, gas movement is predominantly by diffusion, which is the slowest among these steps and thus determines the reaction rate. Thus, the extent of the transport effect can be derived from the diffusion laws. According to Schäfer [35], the following Equation (2) gives the amount of substance transported per unit time in a closed system:

(2) $$R_{s}=\frac{n_{s}\cdot A}{t\cdot L}=\frac{i}{j}\frac{DA∆P}{R\bar{T}Lt\sum P}$$

where R_s_ is the transport rate (mol·cm^−2^·h^−1^); t is the duration of transport (h); n_s_ is the moles of Me(s) transported, *A* and L are the cross-sectional area (cm^2^) and length (cm) of the diffusion path, respectively, and *t* is the duration of transport (h); i and j are the stoichiometric coefficients in the transport of Equation (1); D is the diffusion coefficient; ΔP is the partial pressure difference in substance MeI_x_ between the source and the deposition region (Pa); ΣP is the total pressure (Pa); $\bar{T}$ is the optimum transport temperature along the diffusion path (K), which can be selected as the average of source and the deposition region (K) $\bar{T}\approx (T_{1}+T_{2})/2$, and be preliminary estimate for the experiment with the help of standard data given for 298 K, by Equations (3) and (4) at the equilibrium constant K = 1:

(3) $$LnK=-\frac{{∆}_{r}H_{T}^{0}}{RT}+\frac{{∆}_{r}S_{T}^{0}}{R}$$

(4) $$\bar{T}=-\frac{{∆}_{r}H_{T}^{0}}{{∆}_{r}S_{T}^{0}}$$

In practice, the transport reaction temperature used in experiments is typically higher than the calculated temperature to avoid the possible condensation of reaction products at that calculated temperature.

## 3. Experimental

### 3.1. Raw Materials and Equipment

**Figure 2:**
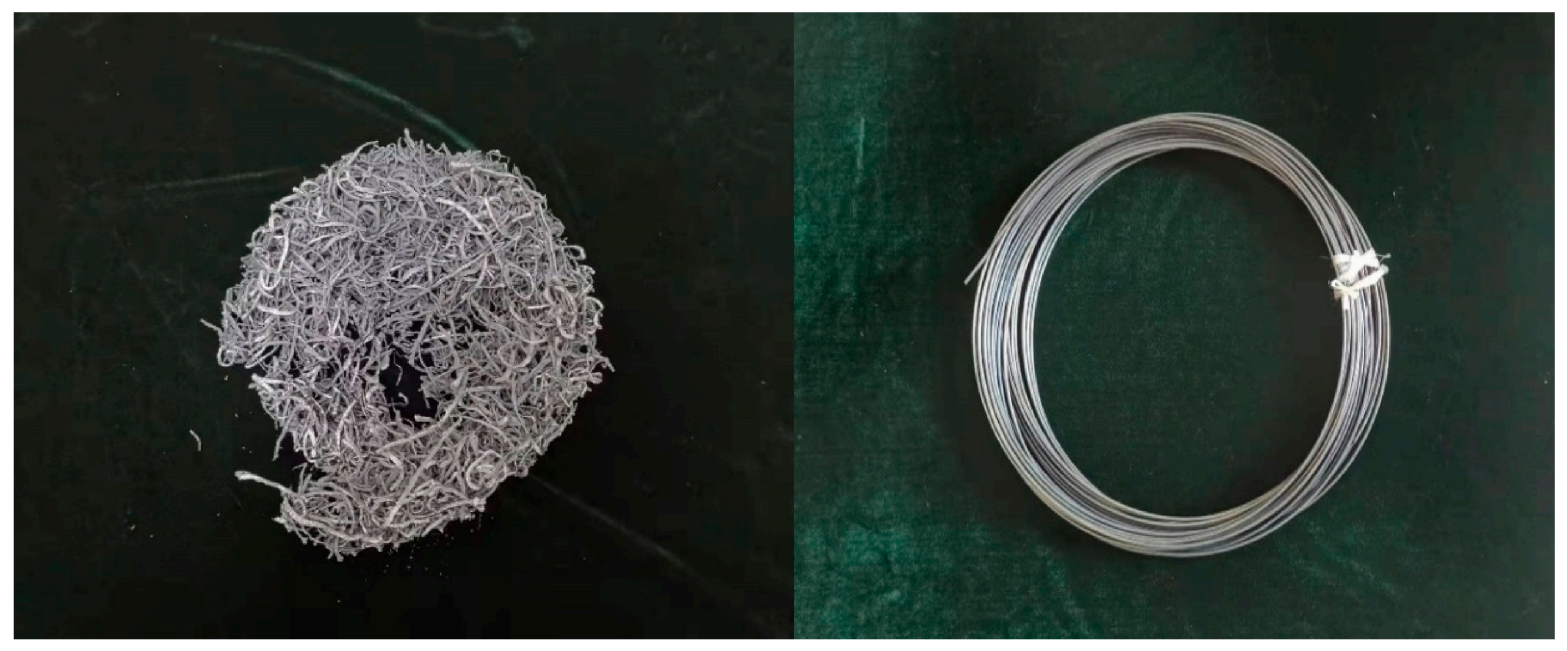
Photos of experimental raw materials.

The main experimental materials included: (1) vanadium granules or chips, bought from Ningxia Orient Tantalum Industry Co., Ltd. (Shizuishan, China), prepared by crushing or turning a vanadium ingot that conforms to grade V-4 of GB/T 4310-2016 [36], used as the crude raw metal; (2) spherical elemental iodine (~99.8 wt.% purity) as the transport agent, bought from ACF Mineral S.A; and (3) vanadium wire, bought from Ningxia Orient Tantalum Industry Co., Ltd., with a diameter of about 3 mm (~99.0 wt.% purity) as the deposition substrate, whose impurity contents also satisfy the V-4 grade of GB/T 4310-2016, with the impurity element contents of the vanadium and iodine given in Table 1 and Table 2, respectively. Photographs of the actual vanadium chips and vanadium filament are shown in Figure 2.

The granular vanadium has a particle size of 5–20 mm, and the vanadium chips have dimensions of approximately (3~20) × (3~5) × (0.5~2) mm. They were sequentially treated by acid leaching, alkaline washing, and water rinsing to remove surface-adsorbed impurities and oil contamination, and then dried in a low-temperature oven or at room temperature to remove surface-adsorbed moisture before use.

**Figure 3:**
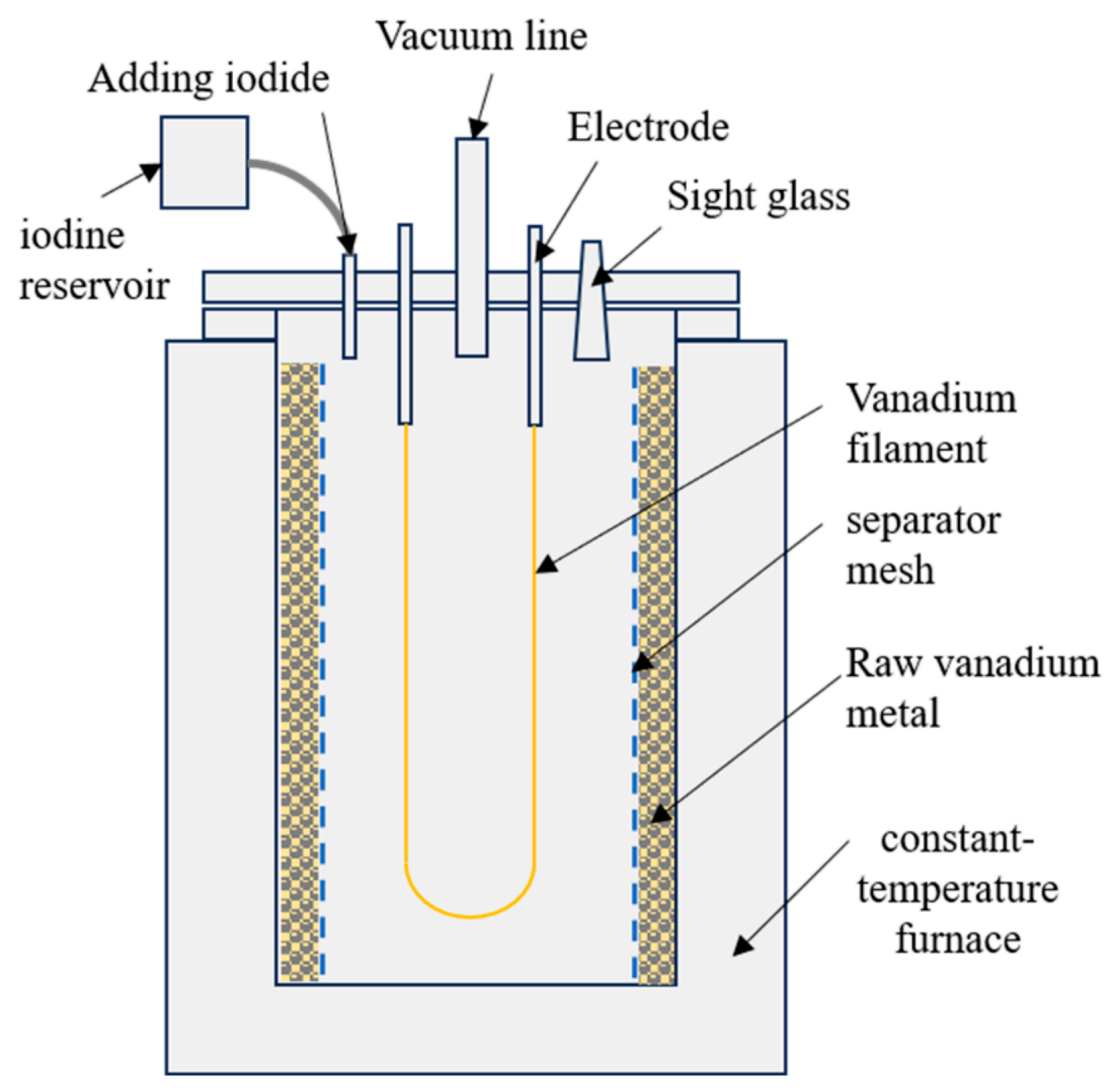
Schematic illustration of the self-made iodide device, adapted from Refs. [37,38,39].

The self-made iodide equipment was adopted [37,38,39], and is shown in Figure 3. The self-made reactor was placed in a constant-temperature furnace equipped with heating and cooling functions. A material isolation mesh was installed inside the reactor, and the crude vanadium metal raw material was placed in the gap between the material isolation mesh and the inner wall of the reactor. The reactor lid was provided with: (1) a vacuum port for connecting to a vacuum system; (2) an observation port for observing the internal reaction during the experiment; (3) current-carrying electrodes, to which the deposition parent filament was welded; during the iodization process, the current and voltage were applied and adjusted to maintain the temperature of the deposition parent filament; and (4) an iodine injection port, through which elemental iodine from the iodine reservoir was injected into the sealed reactor after the experimental conditions were reached inside the reactor.

### 3.2. Experimental Procedure

A single-variable method was adopted in the experiment, and the specific experimental parameters are summarized in Table 3. The K value, T~K = UI^1/3^, where U and I are the voltage and current applied to the deposition filament, respectively, is an empirical parameter related to the current and voltage, used to characterize the temperature of the deposition filament [40]. The initial iodine amount represents the relative magnitude of the total system pressure. Different iodine loadings, after vaporization at the experimental temperature, yield different total pressures in the closed reactor. This is intended to analyze the qualitative relationship between transport behavior and total pressure, rather than to establish a quantitative correspondence with a specific iodine loading or measured pressure. The different raw packing configurations are employed to approximately characterize the different transport distances and incidence angles when the gaseous species formed in the source region diffuse to the deposition filament at similar distances.

**Figure 4:**
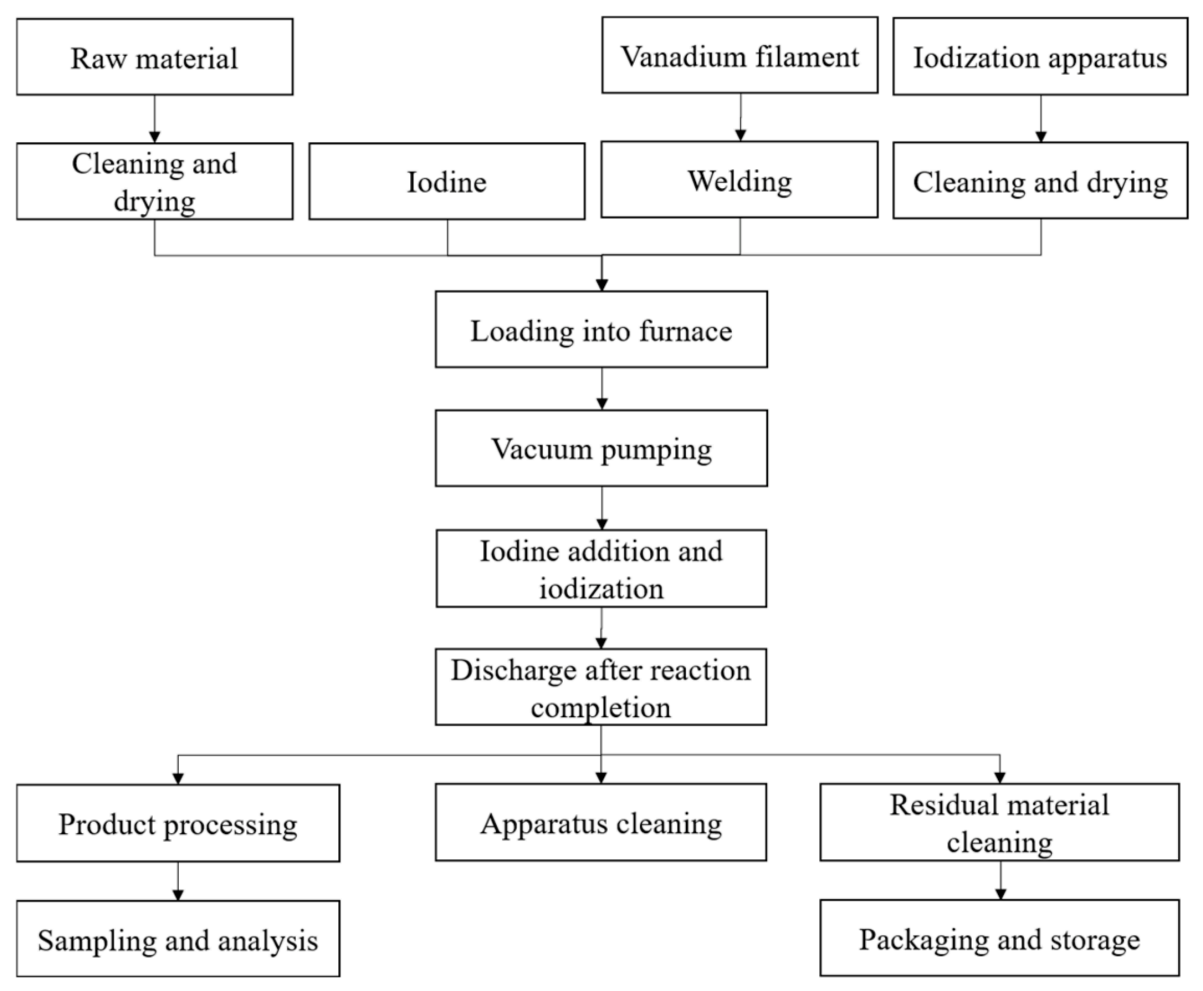
Process flow diagram of the iodide refining process for vanadium.

The process flow diagram is presented in Figure 4, and the detailed experimental procedure is as follows:

(1) Loading. The crude vanadium metal raw material was placed into the gap between the material isolation mesh and the inner wall of the reactor. A vanadium metal wire was welded to the lower end of the electrode using an argon arc welding machine. Elemental iodine was sealed in the iodine reservoir.

(2) Vacuum pumping. The furnace lid was assembled and connected to the reactor. The vacuum system was then connected, and the system was evacuated.

(3) Iodine addition. After the vacuum level reached 10^−2^ Pa and the furnace temperature had risen to the preset experimental temperature, elemental iodine from the iodine reservoir was injected into the sealed reactor through the iodine injection port.

(4) Iodization. The current and voltage conditions set for the experiment were applied, and the iodization reaction began. After the reaction was completed, the power supply was switched off. After the reaction apparatus had naturally cooled to room temperature, argon was introduced until atmospheric pressure was reached, and the vanadium crystal rod was removed.

(5) Analysis. The reaction products and residues were weighed, and the crystal rod was cut and sampled for analysis.

### 3.3. Analytical Methods

The impurity contents in the product were analyzed by the methods as follows: the oxygen (O) and nitrogen (N) contents were analyzed using the inert gas pulse infrared thermal conductivity method; the carbon (C) content was analyzed using the high-frequency combustion infrared absorption method; and the contents of metallic impurities were analyzed using inductively coupled plasma atomic emission spectroscopy (ICP-AES) and inductively coupled plasma mass spectrometry (ICP-MS). All elemental content analyses were carried out by Guobiao (Beijing) Testing & Certification Co., Ltd. following standard analytical procedures.

## 4. Results and Discussion

### 4.1. Grain Morphology and Purity of Vanadium Crystal Bar

**Figure 5:**
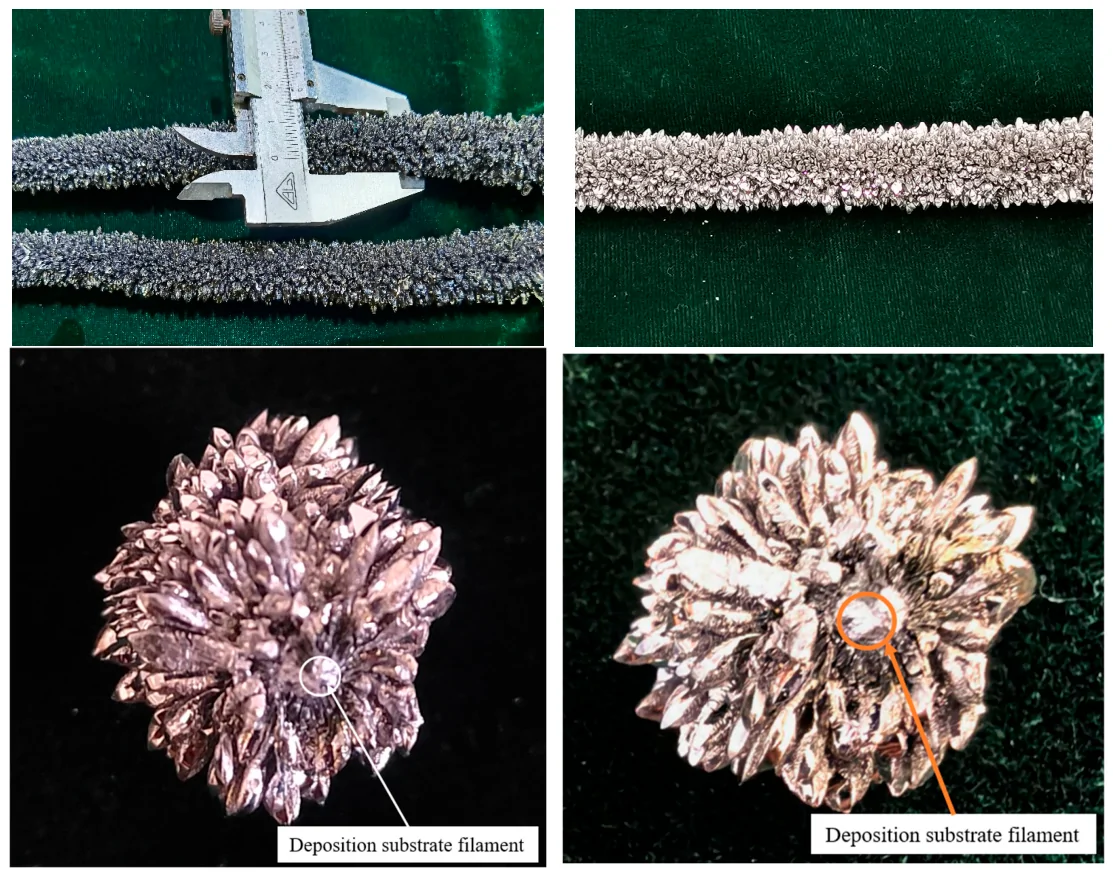
The photos of the crystal vanadium rod.

As shown in Figure 5, the vanadium crystal bar reaches a diameter of approximately 20 mm, with individual grains exhibiting well-defined macroscopic grain boundaries. The grains grow radially outward from the filament surface, and their size increases significantly with increasing radial distance. Based on these macroscopic observations, we propose a mechanistic hypothesis for the grain selection and growth behavior, rather than a proven mechanism. It is hypothesized that during the initial stage of vapor deposition, vanadium atoms nucleate randomly on the filament surface, with a strong scatter in crystallographic orientation, resulting in a large number of fine grains with diverse orientations.

It is further hypothesized that, during the iodide refining process, gaseous VI_2_ molecules are transported from the source zone to the deposition surface, with their macroscopic motion directed radially towards the filament surface (i.e., perpendicular to the iso-concentration surfaces around the cylindrical filament). If the preferential growth direction of an initially nucleated grain coincides with the radial direction, the surface normal of the grain tip would align with the incident atomic flux (i.e., incidence angle θ ≈ 0), yielding the maximum atomic capture cross-section and thus the highest radial growth rate. If the preferential direction deviates from the radial orientation, the angle between the surface normal and the incident flux would increase, reducing the effective capture cross-section and consequently the growth rate.

Under this hypothesis, grains with favorable orientations would gradually overshadow those with less favorable orientations. Meanwhile, adjacent grains sharing the same favorable orientation, having a radial growth advantage, would experience inhibited lateral growth due to impingement of adjacent grain faces, further reinforcing their radial growth dominance. As the favorably oriented grains continue to grow radially, their lateral growth space would expand, and the grain tips would develop into protruding fronts (the rice-grain-shaped tips shown in Figure 5). These protruding tips would physically shield the less favorably oriented grains behind or beside them, placing them in “valley” regions where the effective incidence angle of the atomic flux increases sharply and the direct atomic flux decreases significantly.

It should be emphasized that the above interpretation is based solely on macroscopic photographs and should be regarded as a mechanistic hypothesis. Direct crystallographic evidence is not available in the present study. To confirm the preferred orientation, the competition between differently oriented grains, and the alignment of growth with the flow field, further characterization is required, such as electron backscatter diffraction (EBSD), X-ray diffraction (XRD) texture analysis, or metallographic examination of suitably oriented cross-sections. Such analyses will be specifically discussed in another manuscript by the authors that is currently in preparation for publication.

The impurity element contents in the vanadium crystal bar obtained from the verification experiments are compared with those in the raw material in Table 4. As shown in Table 4, compared with the raw material, the contents of impurity elements Fe and O in the vanadium crystal bar obtained by iodide refining were both reduced by one order of magnitude, falling to 0.025–0.033 wt.% and less than 0.01 wt.%, respectively; the impurities Cr, Al, and Si were reduced to <0.001 wt.%, <0.001 wt.%, and <0.002 wt.%, respectively.

Considering that: (1) the vanadium filament employed as the deposition substrate initially contained a non-negligible amount of Fe, which contributed unfavorably to the overall Fe content of the final crystal bar; and (2) the corrosive reaction between the high-temperature iodine vapor and the steel reactor parts introduced secondary contamination, it is reasonable to expect that, under optimized conditions, iodide refining could produce vanadium with an even lower Fe content than that achieved in the present experiments. Thermodynamically, iron iodide is less stable than the other metal iodides present, which implies a possible co-deposition of iron atoms with vanadium. To solve this problem, the reactor material should be changed or modified. Possible strategies include: (i) using a quartz or ceramic liner to isolate the iron-containing reactor wall from the iodine vapor; (ii) minimizing the contact between high-temperature iodine vapor and steel components; and (iii) employing a reactor material that forms stable iodides with low volatility. These measures will be systematically examined in future work.

### 4.2. Effect of System Temperature on Deposition Rate

**Figure 6:**
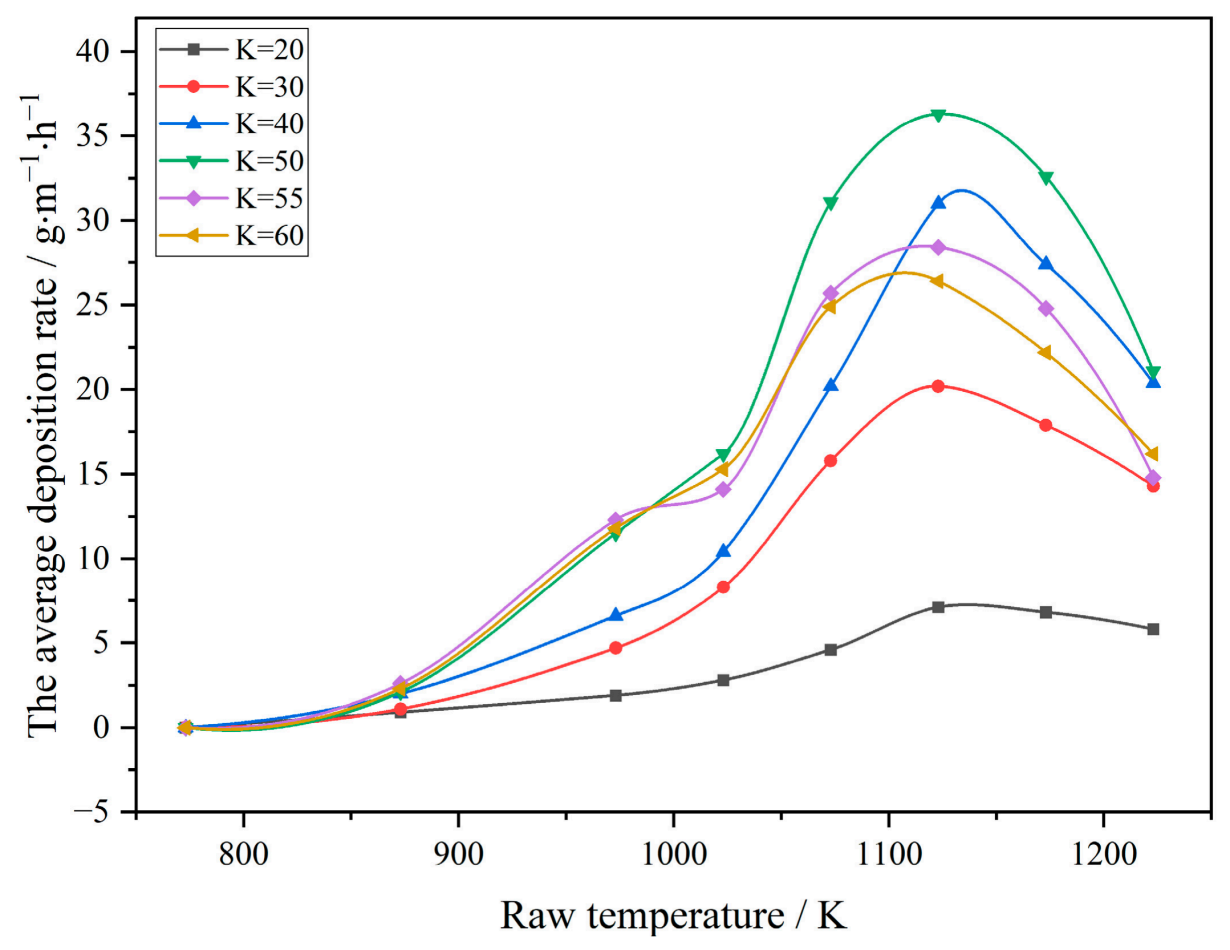
The average deposition rate as a function of different temperatures at different K values (The average deposition rate was calculated from the total mass gain divided by the total reaction time; error bars are not applicable).

During the experiments, the instantaneous deposition rate of vanadium on the filament was not constant, as indicated by the varying rates of change in filament conductivity. Therefore, the average deposition rate was defined as the mass gain of the crystal bar divided by the total reaction time. As shown in Figure 6, under different K values, the average deposition rate exhibits a trend of first increasing and then decreasing with rising source-zone temperature, although the peak temperature and peak rate vary significantly among the curves. Within a certain temperature range, the partial pressure of gaseous VI_2_ in the source zone increases with temperature, enlarging the partial pressure difference between the source and deposition zones, which in turn enhances the driving force for VI_2_ transport and the migration rate toward the deposition zone. However, excessively low source temperatures limit the kinetics of the iodine-vanadium reaction, restraining the actual formation rate of VI_2_. As the temperature rises, although the equilibrium constant decreases, the reaction rate constant increases exponentially according to the Arrhenius law. The competition between these two opposing effects results in an optimal intermediate temperature at which the VI_2_ generation flux reaches a maximum, thereby supplying the deposition zone with the most abundant gaseous VI_2_ species.

At low K values (K < 30), the filament temperature in the deposition zone is relatively low, suppressing the decomposition and deposition of VI_2_ on the filament surface. In this case, raising the source-zone temperature contributes little to increasing the VI_2_ supersaturation at the deposition zone; thus, the average deposition rate increases only slowly with temperature, and the peak rate remains significantly lower than that at higher K values. When K exceeds 40, the deposition zone reaches a more favorable temperature for nucleation and growth. Under these conditions, the VI_2_ supersaturation at the filament surface and the sustained high partial pressure supply from the source zone become the key factors governing rapid crystal growth. Consequently, the average deposition rate becomes more sensitive to temperature variations: it increases roughly linearly at source temperatures below approximately 1050 K, rises sharply above 1050 K, and reaches a maximum at about 1150 K. Beyond this point, the partial pressure increase in VI_2_ in the source zone slows down, while its consumption due to rapid decomposition and deposition at the deposition zone intensifies, leading to a narrowing of the partial pressure difference between the two zones and a consequent rapid decline in the average deposition rate.

**Figure 7:**
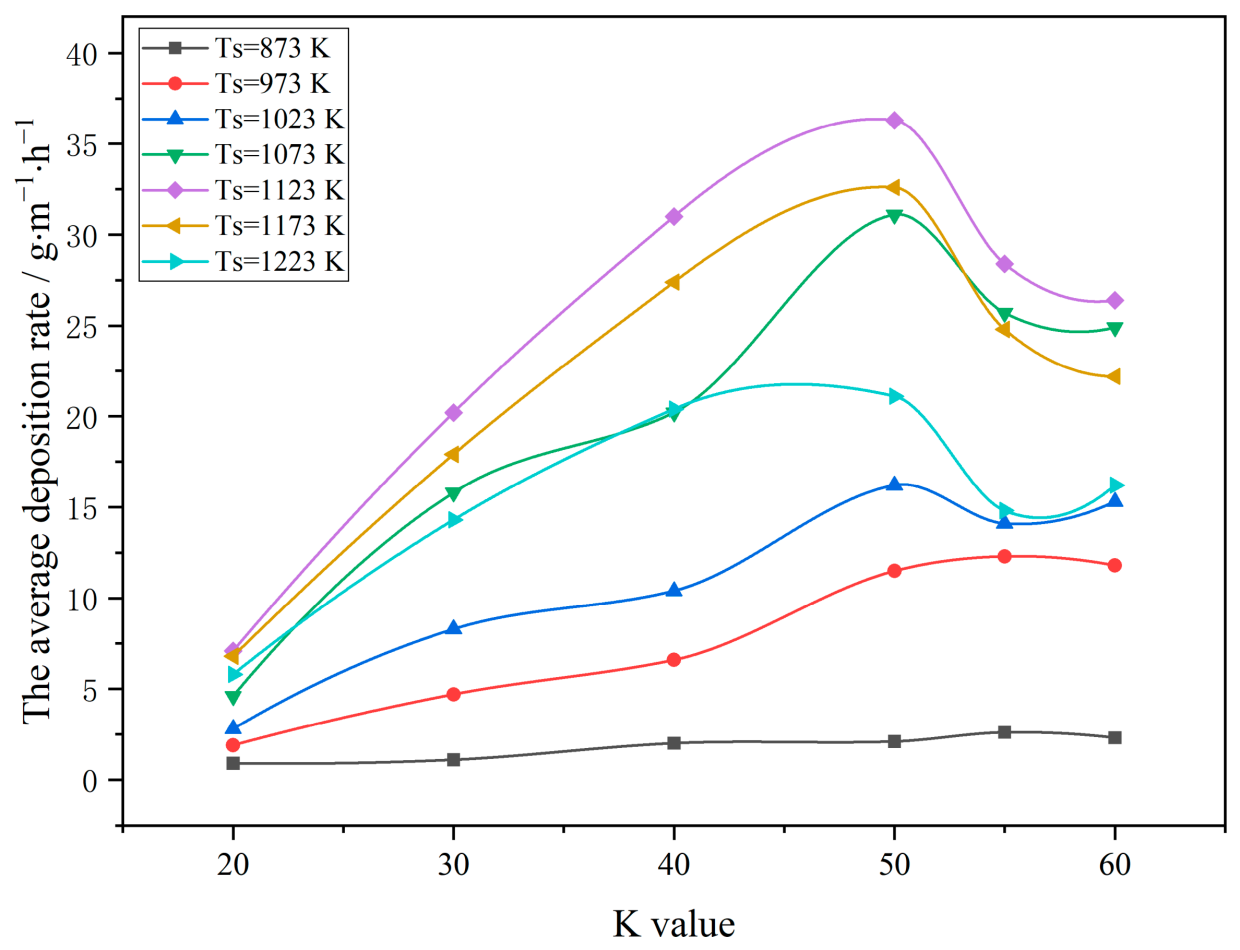
The average deposition rate as a function of different K values at different temperatures (The average deposition rate was calculated from the total mass gain divided by the total reaction time; error bars are not applicable).

As shown in Figure 7, the average deposition rate exhibits a bell-shaped distribution with increasing K value: it first rises, reaches a maximum, then declines and gradually levels off. At low source temperatures (<1023 K), the average deposition rate increases very slowly with K, and the peak rate is significantly lower than that observed under higher source-temperature conditions. At source temperatures of 1073, 1123, and 1173 K, the curves display a pronounced peak structure. However, when the source temperature is further increased to 1223 K, the curve falls between those obtained at lower and higher temperatures.

The K value, which characterizes the filament temperature, primarily determines the driving force for VI_2_ decomposition and deposition, as well as the diffusive mass-transfer coefficient. A higher K value accelerates the decomposition and deposition reactions on the filament surface, but simultaneously increases the partial pressure and back-flow rate of the I_2_ molecules released during deposition. When the I_2_ back-flow rate becomes sufficiently high, a “convective back-flow” effect develops in the gas-phase transport system, reducing the net transport flux of VI_2_ from the source zone to the deposition zone, and consequently leading to a decrease in the average deposition rate. The curves for K = 55 and K = 60 are noticeably lower than those for K = 50, more clearly illustrating this transport phenomenon (Figure 6).

These results indicate that there exists an optimal matching relationship between the source temperature and the deposition temperature. The source temperature determines the VI_2_ supply flux to the deposition zone through its influence on both the equilibrium and kinetics of VI_2_ formation; the deposition temperature governs the capture and incorporation efficiency of the VI_2_ species transported from the source zone, by affecting the decomposition/deposition reaction rate, diffusion coefficient, and back-flow intensity. Thus, the overall gas-phase transport rate is largely governed by the average system temperature.

### 4.3. Effect of Initial Iodine Amount on Deposition Rate

The reaction duration was determined according to the following criteria: from the completion of iodine introduction until (1) the rate of change in filament conductivity dropped to a negligibly small value (i.e., virtually no further change), at which point the reaction was terminated; or (2) the reaction was prematurely interrupted (e.g., due to filament burnout). In practice, the average duration of 2–3 valid runs was used.

**Figure 8:**
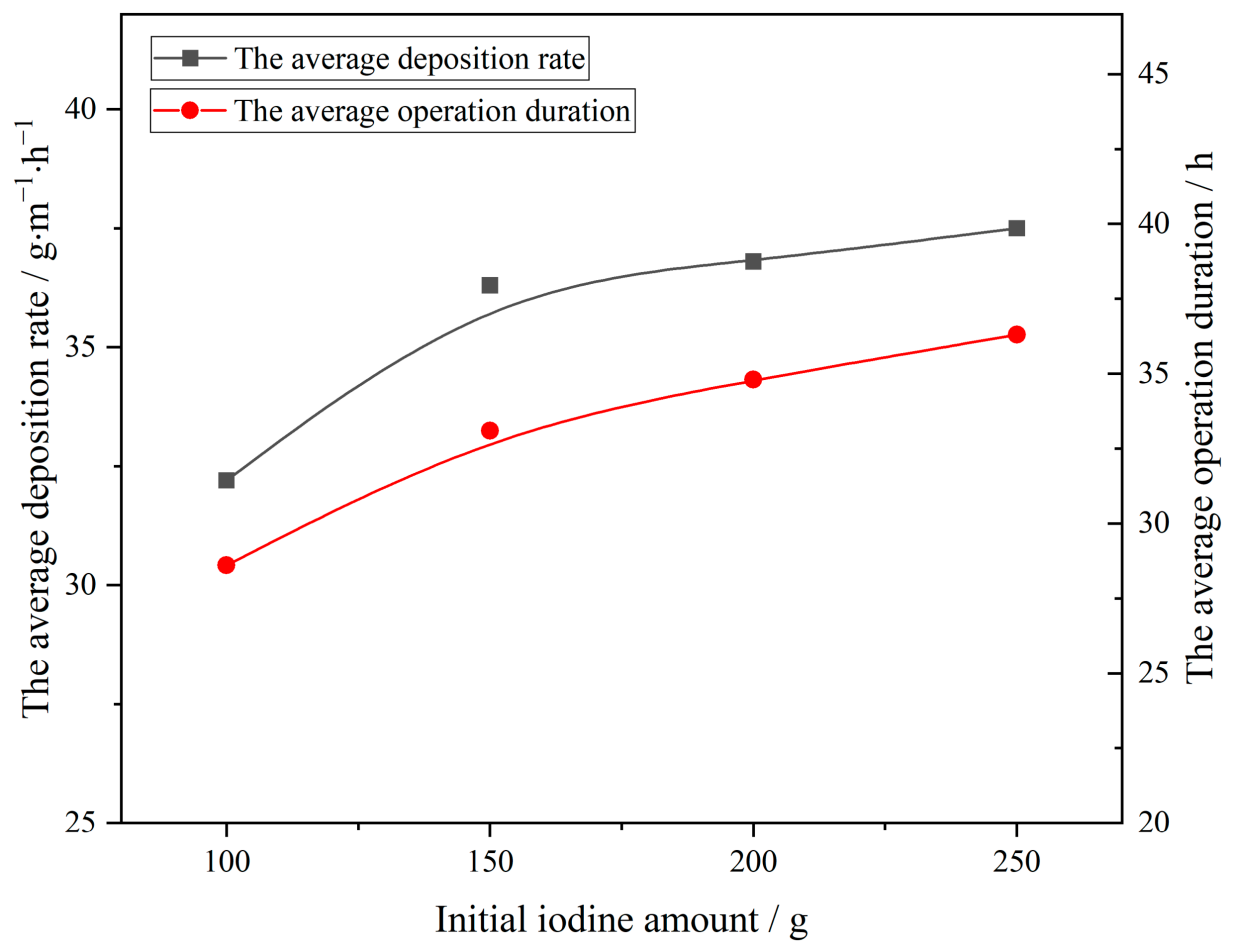
The effect of initial iodine amount on average deposition rate and reaction duration (The average deposition rate was calculated from the total mass gain divided by the total reaction time; error bars are not applicable).

Figure 8 shows the effects of total system pressure (iodine loading) on both the average deposition rate and the average reaction duration at the fixed conditions of raw temperature at 1123 K and K = 50. As shown in Figure 8, both quantities increase only slightly with increasing initial iodine loading, and the overall variation remains relatively limited. Combining the preceding two-dimensional flow field simulation, it is known that at low total system pressures, mass transport is dominated by diffusion, and the transport rate depends primarily on the transport distance of gaseous species, decreasing with increasing distance.

Because the raw vanadium chips were in a fluffy state, the total amount of vanadium that could be loaded into the reactor was limited. Consequently, the initial iodine loading was relatively small, and the corresponding initial total pressure remained in a low range. Within this range, no significant variation in average deposition rate or reaction duration was observed. Nevertheless, as the iodine loading increased, the molar concentration of iodine participating in the reaction rose, which elevated the equilibrium partial pressure of gaseous VI_2_ in the source zone to some extent. This, in turn, enhanced the transport rate and flux of VI_2_ toward the deposition zone, leading to a modest increase in the average deposition rate.

### 4.4. Effect of Raw Material Packing Configuration on the Deposition Process

**Figure 9:**
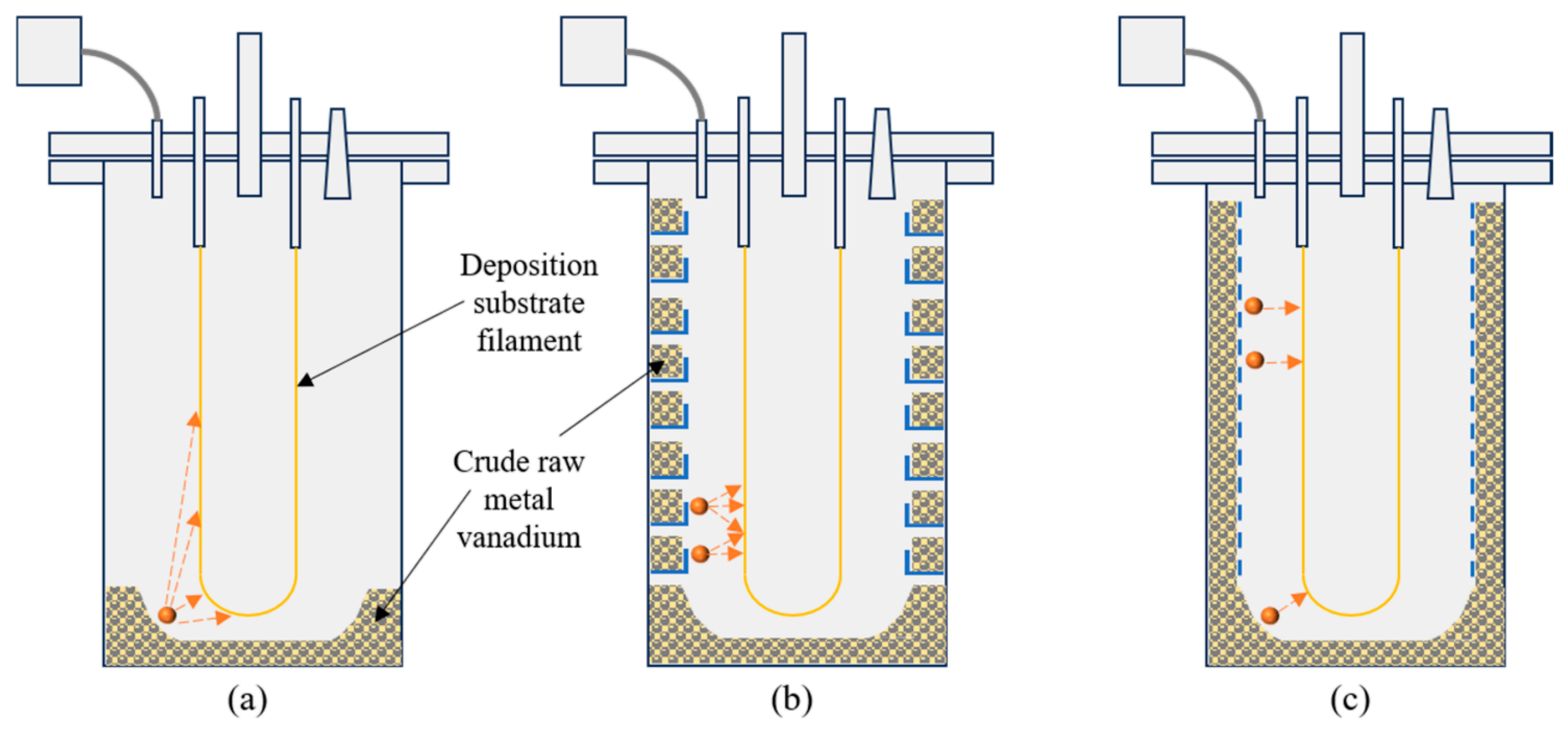
Schematic illustration of raw material arrangement. (**a**) bottom loading; (**b**) intermittent filling; (**c**) full loading.

Three different raw material loading configurations were designed, as illustrated in Figure 9. The yellow spheres represent the gaseous species generated in the source zone, and the yellow dashed lines indicate their transport paths and directions.

(1) In configuration Figure 9a, i.e., bottom loading, gaseous VI_2_ molecules are generated only at the bottom of the reactor. When these molecules travel toward the deposition filament located above, their transport path (i.e., the incidence path of vanadium atoms) forms a relatively large angle with the surface normal of the grain fronts on the distal part of the filament. Moreover, this incidence angle θ increases progressively toward the upper side of the filament. (2) In configuration Figure 9b, i.e., segmented (intermittent) loading, the raw material is packed intermittently along the axial direction of the reactor wall, leaving unfilled gaps. In these gap regions, no atomic flux is directly incident normal to the filament surface. Instead, the atomic flux arriving at the filament surface in these regions is supplied by the gaseous species generated from the packed zones above and below, with the incidence angle θ ranging between 0° and 90°. (3) In configuration Figure 9c, i.e., full loading, the raw material completely fills the bottom and inner wall surfaces of the reactor. In this case, every position along the deposition filament receives a uniform atomic flux, with the flux direction parallel to the surface normal of the grain fronts on the filament (i.e., incidence angle θ = 0°).

**Figure 10:**
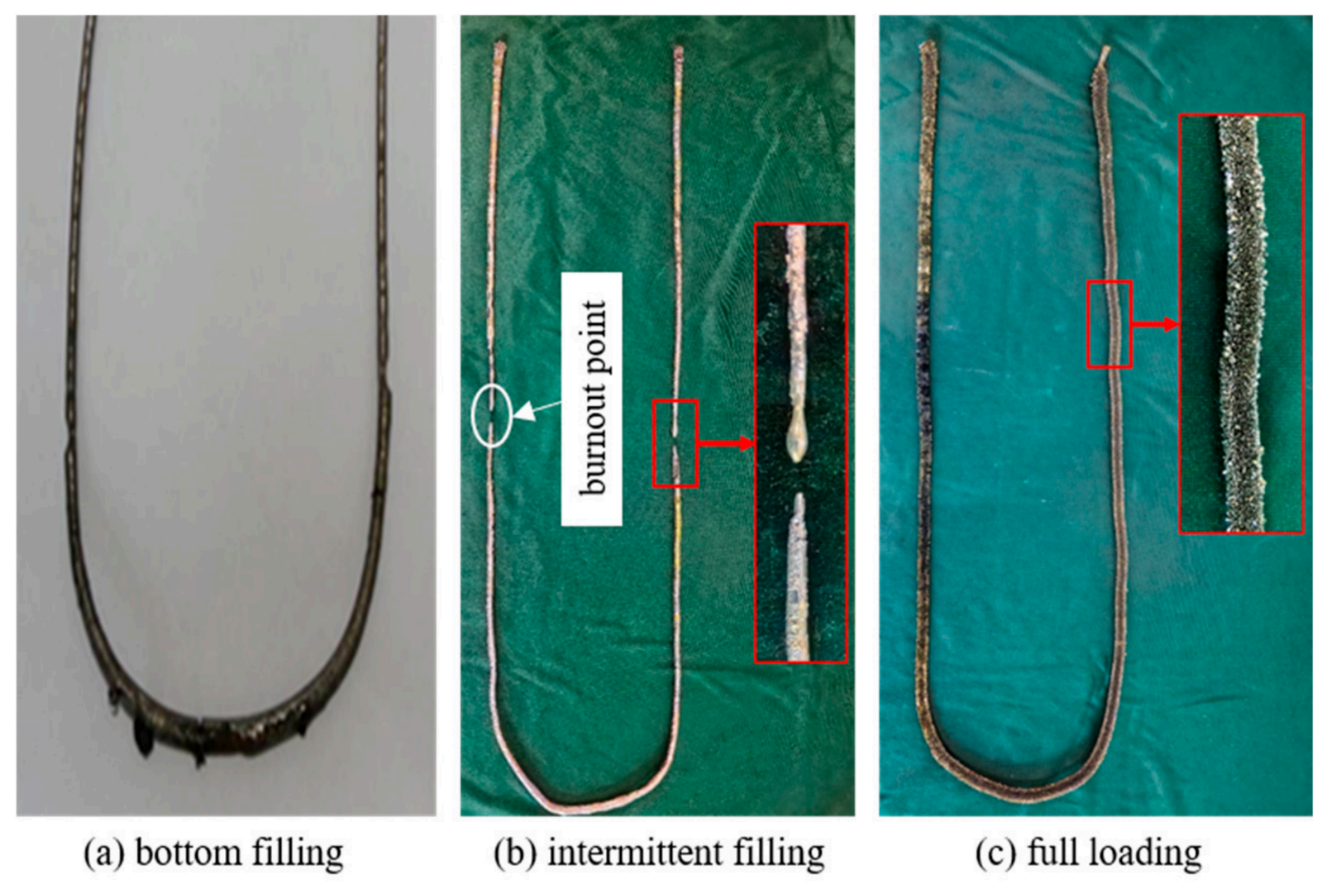
The effect of raw material arrangement on the deposition process.

In the bottom loading configuration (Figure 10a), the grain fronts on the upper part of the filament, which is farther from the source material, receive virtually no atomic flux parallel to their surface normal. In addition, the transport path of gaseous VI_2_ molecules are considerably longer than that near the bottom, resulting in a lower VI_2_ partial pressure and reduced supersaturation in this region, which severely suppresses the further growth of the initial grains. Consequently, the final crystal bar exhibits a pronounced tapered morphology, with the bottom diameter significantly larger than the upper portion, and notable diameter variations along its length.

In the segmented loading configuration (Figure 10b), the transport distance from the source zones to the adjacent filament surface is substantially shorter than in the bottom loading case, and the variation in transport distance among different positions is relatively small. In the gap regions corresponding to unfilled zones, although the filament surface does not receive a completely normal atomic flux, the limited gap size allows the VI_2_ molecules generated from the packed zones above and below to still provide sufficient atomic flux with relatively small incidence angles to sustain the growth of preferentially oriented grains. However, because the gaseous species flux near the filament surface in these gap regions relies entirely on the adjacent packed zones, the grain growth rate in these regions is comparatively slower. As the deposition proceeds, the competitive growth mechanism further restricts grain growth in these regions, eventually leading to stagnation. These slowly growing or arrested regions develop smaller diameters compared to the rest of the bar, becoming weak points that are prone to burnout or fracture.

In the full loading configuration (Figure 10c), the transport distance and net flux of VI_2_ molecules are essentially uniform along the entire filament surface, and each grain front receives a nearly uniform atomic flux from all incidence angles. As a result, the final vanadium crystal bar exhibits good uniformity in both diameter and grain morphology along its entire length.

## 5. Numerical Simulation

### 5.1. Physical Model and Boundary Conditions

**Figure 11:**
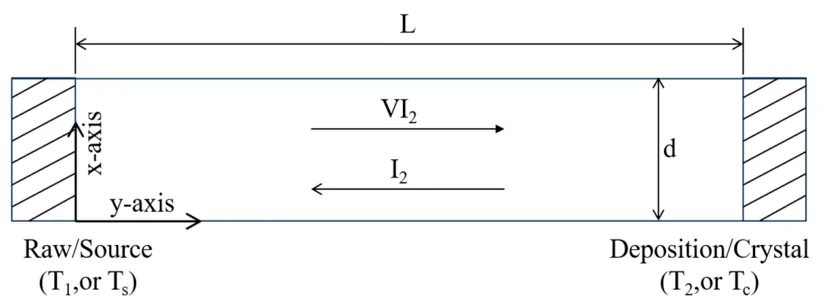
Schematic diagram of the horizontal rectangular enclosure physical model.

The actual experiment was conducted in a cylindrical enclosed reactor, with the raw material and the deposition substrate arranged near the inner wall of the reactor and at the central position of the reactor, respectively. In other words, along the radial direction of the reactor, the raw material was uniformly distributed at equal distances around the deposition substrate. A horizontal rectangular enclosure with two vertical walls was adopted as the physical model (Figure 11), obtained by simplifying the vertical mid-plane of the actual three-dimensional cylindrical ampoule to a two-dimensional symmetric representation. It is assumed that the width L of the enclosure is much greater than its height d, allowing the effect along the width dimension to be neglected.

The temperatures in the raw material zone and the deposition zone are approximately 1123 K and 1673 K, respectively [28]. In this temperature range, the system space is primarily composed of a significant amount of three vapor-phase species: I_2_, I and VI_2_. Thus, the following main reactions will occur simultaneously in the system:

(5) V(s)+I2(g)⇌VI2(g),   K1=PVI2PI2

(6) I2(g)⇌2I(g),   K2=PI2PI2

Since the initial system contains only I_2_, according to the conservation of iodine atoms, the following relation is obtained at equilibrium:

(7) $$P_{I_{2}}+P_{VI_{2}}+\frac{1}{2}P_{I}=P_{0}$$

Due to the large temperature difference between the two zones, the density of the vapor species in the chamber varies significantly with spatial position. To minimize modeling errors, general conservation equations that account for the compressibility effect and the variation in thermophysical parameters with temperature and pressure are adopted for the analysis. The four partial differential equations derived from the conservation laws can be expressed by the following generic equation:

(8) $$\nabla \cdot \left(\rho \vec{u}\phi \right)=\nabla \cdot \left(\Gamma \nabla \phi \right)+S$$

where ∇ = (∂/∂x, ∂/∂y) is the gradient operator, and ∇· denotes the divergence operator, $\vec{u}$ is the velocity vector, ϕ represents the corresponding variable in each conservation equation, Γ is the diffusion coefficient, and S is the source term. In each numerical interpolation step, all other terms in the above equation are assumed to remain constant. The variables and constants corresponding to the different conservation equations for Equation (8) are listed in Table 5. In Table 5, the source term for the mass component conservation equation is set to zero. The pressure diffusion term is neglected because the total pressure is assumed to be spatially uniform in the present model, consistent with the low-pressure conditions. It should be noted that the total pressure itself is still treated as a global parameter, and its variation alters the gas density, diffusion coefficient, and buoyancy intensity, thereby affecting the flow pattern and transport regime.

During the reaction process, since I_2_ species are not inert but undergo dynamic reaction and exchange at both the source and deposition interfaces, the boundary conditions for velocity, temperature, and species mass fractions in the model can be expressed as follows (for simplicity, ω is used uniformly to denote $\omega_{{VI}_{2}}$):

(1) Source end:

(9) $$u\left(0,y\right)=-\frac{D_{ij}}{M_{{VI}_{2}}/M_{V}-\omega (0,y)}\frac{\partial \omega (0,y)}{\partial x}\;u\left(0,y\right)=0\;T\left(0,y\right)=T_{s}\;\omega \left(0,y\right)=\frac{M_{{VI}_{2}}P_{{VI}_{2}}(0,y)}{M_{{VI}_{2}}P_{{VI}_{2}}\left(0,y\right)+M_{I}P_{I}\left(0,y\right)+M_{I_{2}}P_{I_{2}}(0,y)}$$

(2) Crystal end:

(10) $$u\left(L,y\right)=-\frac{D_{ij}}{M_{{VI}_{2}}/M_{V}-\omega (L,y)}\frac{\partial \omega (L,y)}{\partial x}\;v\left(L,y\right)=0\;T\left(L,y\right)=T_{c}\;\omega \left(L,y\right)=\frac{M_{{VI}_{2}}P_{{VI}_{2}}(L,y)}{M_{{VI}_{2}}P_{{VI}_{2}}\left(L,y\right)+M_{I}P_{I}\left(L,y\right)+M_{I_{2}}P_{I_{2}}(L,y)}$$

(3) boundary region:

(11) $$u\left(x,0\right)=u\left(x,d\right)=0\;v\left(x,0\right)=v\left(x,d\right)=0\;T\left(x,0\right)=T\left(x,d\right)=T_{s}-(T_{s}{-T}_{c})\frac{x}{L}\;\omega \left(x,0\right)=\omega \left(x,d\right)=\omega_{s}-(\omega_{s}{-\omega }_{c})\frac{x}{L}$$

where ω_s_ and ω_c_ are the mass fractions at the source and crystal ends, $M_{{VI}_{2}}$, $M_{V}$, $M_{I}$, and $M_{I_{2}}$ are the molecular weights of VI_2_, V, I and I_2_, respectively, and $P_{{VI}_{2}}$, $P_{I}$ and $P_{I_{2}}$ are the partial pressures of VI_2_, I and I_2_, respectively. To solve Equations (8)–(11) simultaneously, the following simplifications are made [33,34]:

(1) Only VI_2_ and I_2_ are considered as the transporting species in the diffusion and thermophysical property calculations, and the contribution of I to diffusion is neglected.

(2) The viscosity of I_2_ is taken to represent that of the VI_2_–I_2_ vapor mixture, avoiding the complexity of multi component viscosity calculations.

(3) The same simplification is applied to thermal conductivity and constant-pressure heat capacity.

(4) Local thermodynamic equilibrium is assumed at both the source and the filament surfaces.

Clearly, the above assumptions have notable limitations, which will be discussed collectively in a dedicated section later in this paper. Under these assumptions, the diffusion coefficient D can be calculated using the relatively simple Gilliland equation [41]:

(12) Dij=4.3×10−7T32(1/Mi+1/Mj)12P(Vi1/3+Vj1/3)2

where the units of D_ij_, P, M_i_, V_i_ are m^2^/s, atm, kg/kmol, and cm^3^, respectively; V_i_ is numerically equal to M_i_/ρ_i_, where ρi denotes the liquid-phase density of species i. The viscosity μ, thermal conductivity k, and constant-pressure heat capacity C_p_ of the nonpolar I_2_ molecules may be obtained from the Chapman–Enskog theory [42]:

(13) $$D_{ij}=6.4167\times 10^{-10}\frac{T^{3/2}}{P}$$

(14) $$\mu =2.6693\times 10^{-26}\frac{{\left(MT\right)}^{1/2}}{\sigma^{2}\Omega_{\mu }}=1.455\times 10^{-6}T^{1/2}$$

(15) $$k=8.3224\times 10^{-22}\frac{{\left(T/M\right)}^{1/2}}{\sigma^{2}\Omega_{\mu }}=1.786\times 10^{-4}T^{1/2}$$

(16) $$C_{p}=\frac{7R}{2M}=114.5$$

where the viscosity μ is in kg/(m · s); M is the molecular weight of iodine, in kg/kmol; T is the average temperature, in K; σ is the collision diameter, in m; and Ω_μ_ is the collision integral. The thermal conductivity k is in W/(m · K), and the constant-pressure heat capacity C_p_ is in J/(kg · K).

And the Grashof number, which characterizes the relative strength of buoyancy to viscous forces, is defined as follows [43]:

(17) $$G_{r}=\frac{g\beta ∆Td^{3}}{v^{2}}$$

where g is the gravitational acceleration (m/s^2^), β is the thermal expansion coefficient (1/K), ΔT is the temperature difference between the source and crystal ends (K), d is the enclosure height (m), and ν is the kinematic viscosity (m^2^/s).

The conservation equations (Equation (8)) are solved using the SIMPLE algorithm program based on the finite volume method and staggered grid, together with the boundary conditions given by Equations (9)–(11), the basic thermophysical parameters listed in Equations (13)–(16), and the equilibrium constants of the reaction equations obtained from thermodynamic calculation software. In the computation, a grid of 40 × 25 is adopted, and the convergence tolerance is set to 1 × 10^−8^. Depending on the specific case, about 6000 to 10,000 iterations are typically required for the program to reach convergence.

### 5.2. Modeling Results and Discussions

#### 5.2.1. Transport Rate as a Function of Different Total Pressure P_0_ and Transport Lengths L

**Figure 12:**
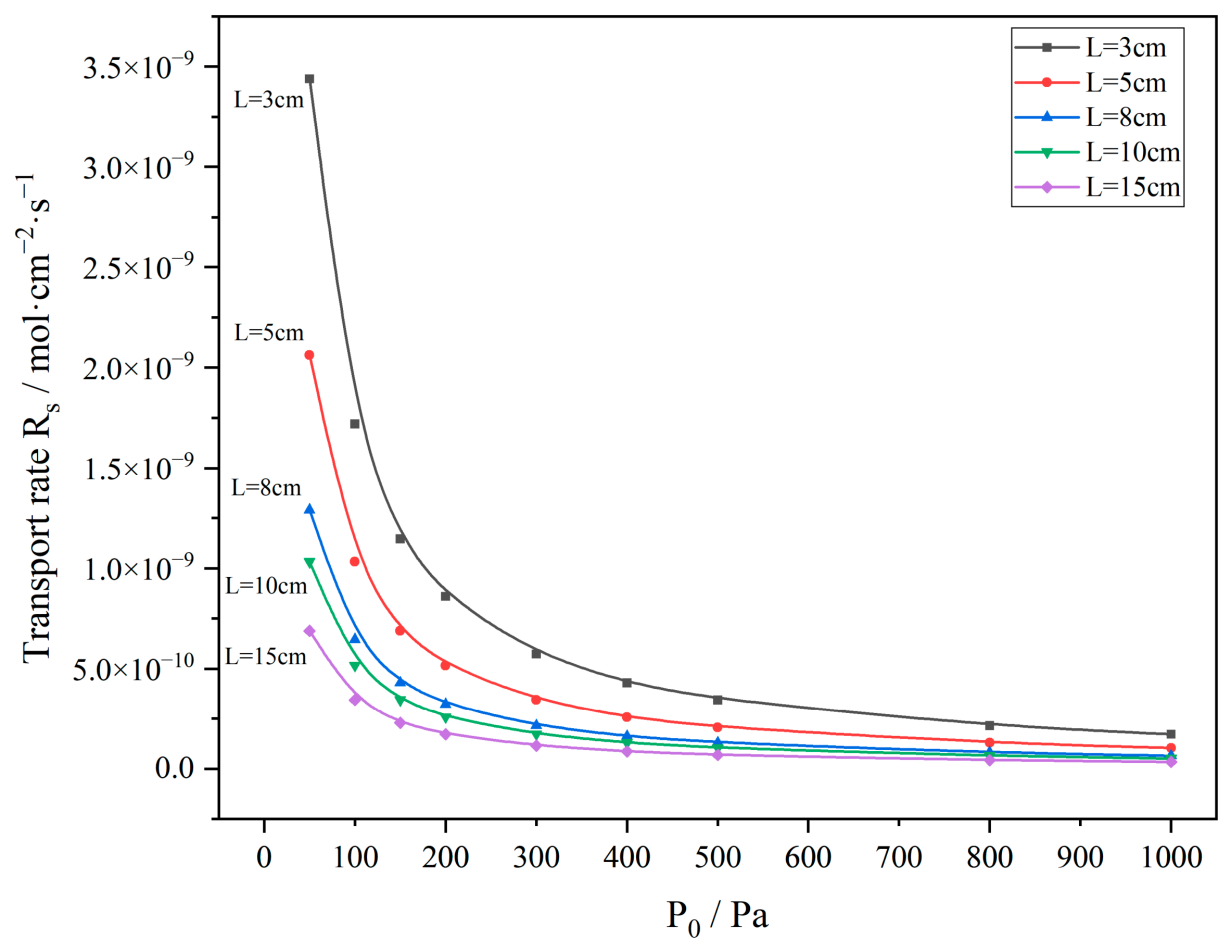
Transport rate as a function of total pressure for different model lengths at fixed temperatures (T_s_ = 1123 K and T_c_ = 1773 K).

The variation in transport rate R_s_ with system pressure P_0_ and at transport length L at fixed temperatures (T_s_ = 1123 K, T_c_ = 1773 K) is depicted in Figure 12. As shown in Figure 12, the transport rate decreases sharply with increasing total system pressure. When the pressure reaches 500 Pa, the transport rate tends to level off and shows little further variation with increasing pressure. At low system pressures, the transport rate decreases significantly with increasing transport distance; however, this decreasing trend becomes less pronounced as the distance increases. Moreover, as the total pressure rises, the differences in transport rate among various transport distances gradually diminish, eventually converging to a relatively narrow range.

This behavior can be attributed to the inverse relationship between the diffusion coefficient and total system pressure, as given by Equation (13), combined with the fact that the partial pressure difference in the transporting species remains essentially constant at a given temperature (since the equilibrium constant is fixed). From the one-dimensional diffusion flux expression (R_s_ = D × ΔP/L), it follows that the transport rate decreases with increasing pressure. As the system pressure continues to rise, the diffusion resistance gradually becomes saturated, and the mass transport becomes governed by coupled diffusion and convection. In this regime, further increases in pressure have only a marginal effect on the density field, and the influence of transport distance L also diminishes. Consequently, the transport rate converges within a limited range at high pressures, reflecting a transition from diffusion-controlled transport at low pressures to convection-enhanced transport at high pressures, with the pressure dependence of the transport rate becoming increasingly weak across different transport distances.

#### 5.2.2. Transport Rate as Function of Temperatures T_s_ and T_c_

**Figure 13:**
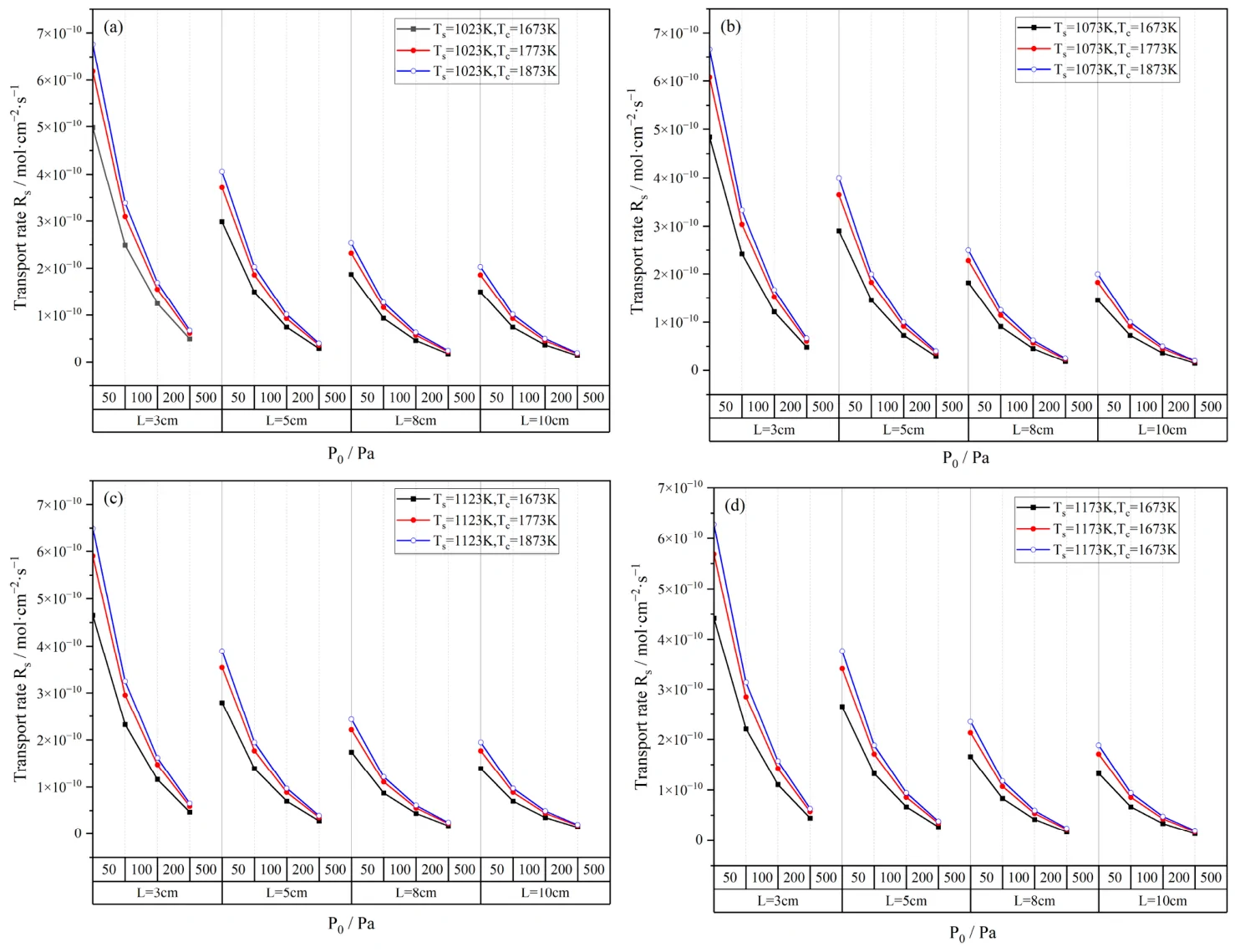
Transport rate as a function of total pressure under different process parameters (T_s_ = 1023, 1073, 1123 and 1173 K; T_c_ = 1673, 1773 and 1873 K; L = 3, 5, 8 and 10 cm). (**a**) Ts = 1023 K; (**b**) Ts = 1073 K; (**c**) Ts = 1123 K; (**d**) Ts = 1173 K.

Figure 13 presents the simulated total mass transport rate as a function of total system pressure P_0_, source and deposition zone temperatures, and transport distance L. Under fixed source and deposition temperatures, the transport rate gradually decreases with increasing total system pressure, and the decreasing trend tends to level off at higher pressures, consistent with the results shown in Figure 12. The temperature effect exhibits a distinct dual competitive mechanism. As the source temperature increases from 1023 K to 1173 K, the reaction equilibrium constant decreases, reducing the partial pressure of gaseous VI_2_ in the source zone and thereby weakening the diffusional driving force. Meanwhile, as the deposition temperature rises from 1673 K to 1873 K, the equilibrium constant further decreases, resulting in an even lower VI_2_ partial pressure at the deposition end. However, for a fixed transport distance L, the transport rate paradoxically increases with temperature. This indicates that, although the VI_2_ partial pressures at both ends decrease with increasing temperature, the partial pressure difference between the two zones actually increases, becoming the dominant driving force for the diffusion of VI_2_ molecules from the source to the deposition zone. This finding is in agreement with the theoretical analysis presented earlier.

Furthermore, the effect of transport distance L on the transport rate follows Fick’s law closely, showing an approximately inverse relationship between the transport rate and the distance. This further confirms that, within the pressure range investigated, diffusion is the primary mechanism controlling mass transport. It should be noted, however, that further increases in either transport distance or system pressure may introduce convective effects, causing the transport rate to deviate from the purely diffusion-based Fickian relationship. In summary, the simulation results clearly reveal the characteristic mass transport VI_2_ system: the transport rate decreases monotonically with increasing total pressure, with the decreasing rate gradually tapering off; it increases with rising temperature due to the enhanced diffusional driving force from an increased VI_2_ partial pressure difference; and it decreases nonlinearly with increasing transport distance. The simulation results are in good qualitative agreement with the analytical solutions derived from the equilibrium-based diffusion theory, which, to some extent, validates the SIMPLE algorithm and the overall simulation procedure.

#### 5.2.3. Flow Pattern, Flow Transition and Flux Transition

**Figure 14:**
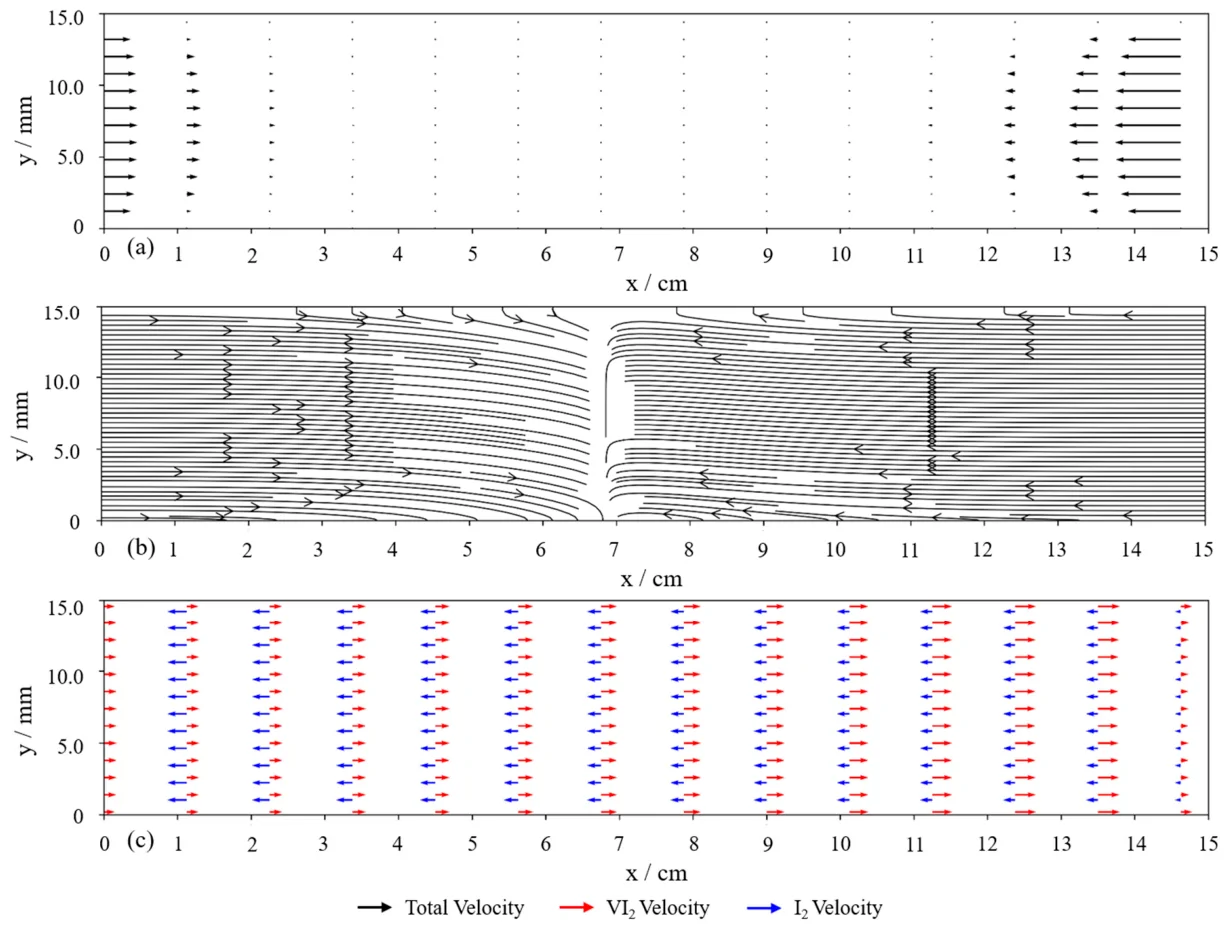
Flow patterns for pure diffusion/advection near the two ends at P_0_ = 300 Pa (T_s_ = 1123 K, T_c_ = 1773 K, L = 15 and d = 1.5 cm). (**a**) Total velocity discrete plot; (**b**) Total velocity streamline plot; (**c**) Component velocity plot.

**Figure 15:**
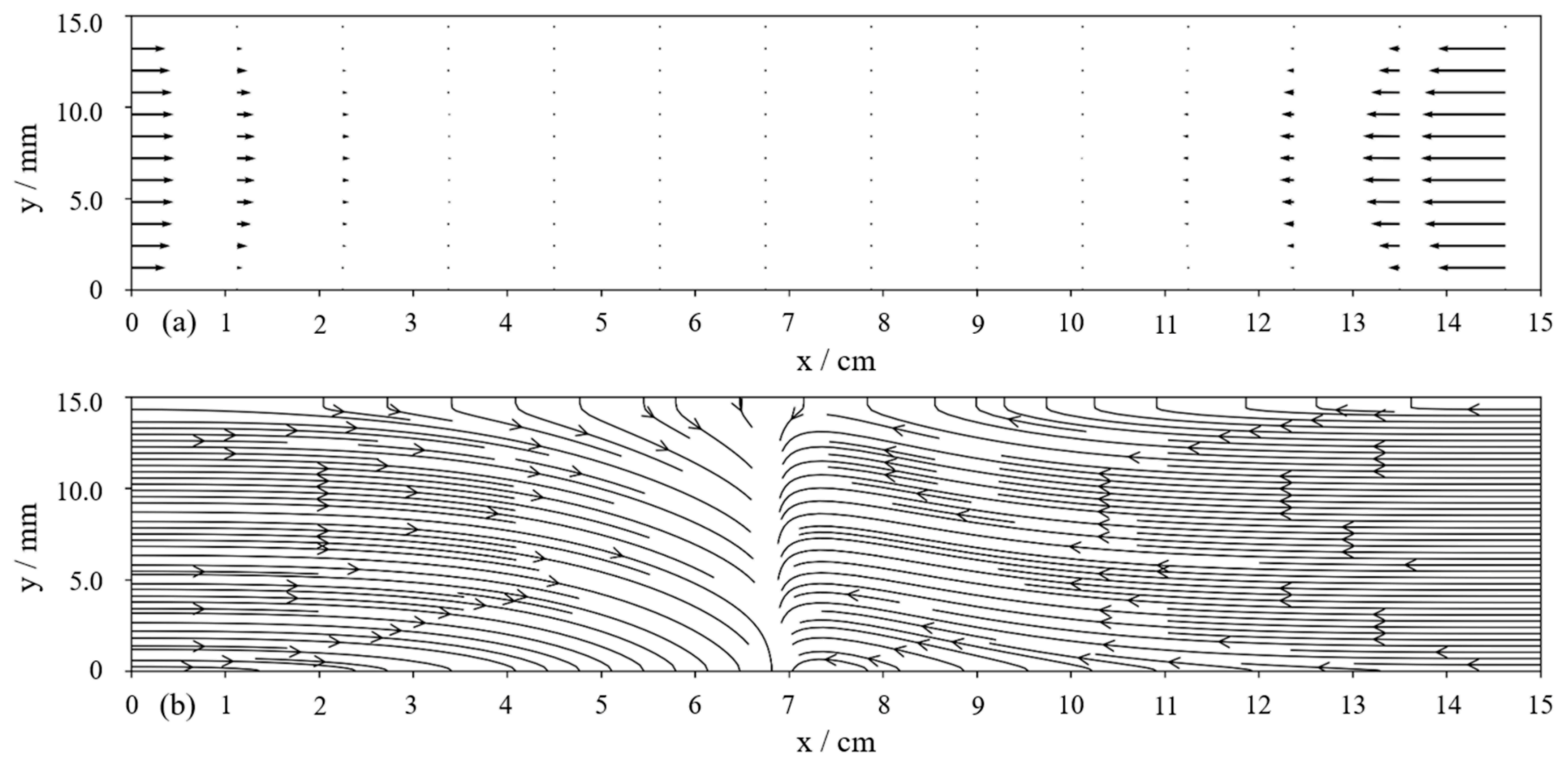
Flow patterns for pure diffusion/advection near the two ends at P_0_ = 500 Pa (T_s_ = 1123 K, T_c_ = 1773 K, L = 15 and d = 1.5 cm). (**a**) Total velocity discrete plot; (**b**) Total velocity streamline plot.

**Figure 16:**
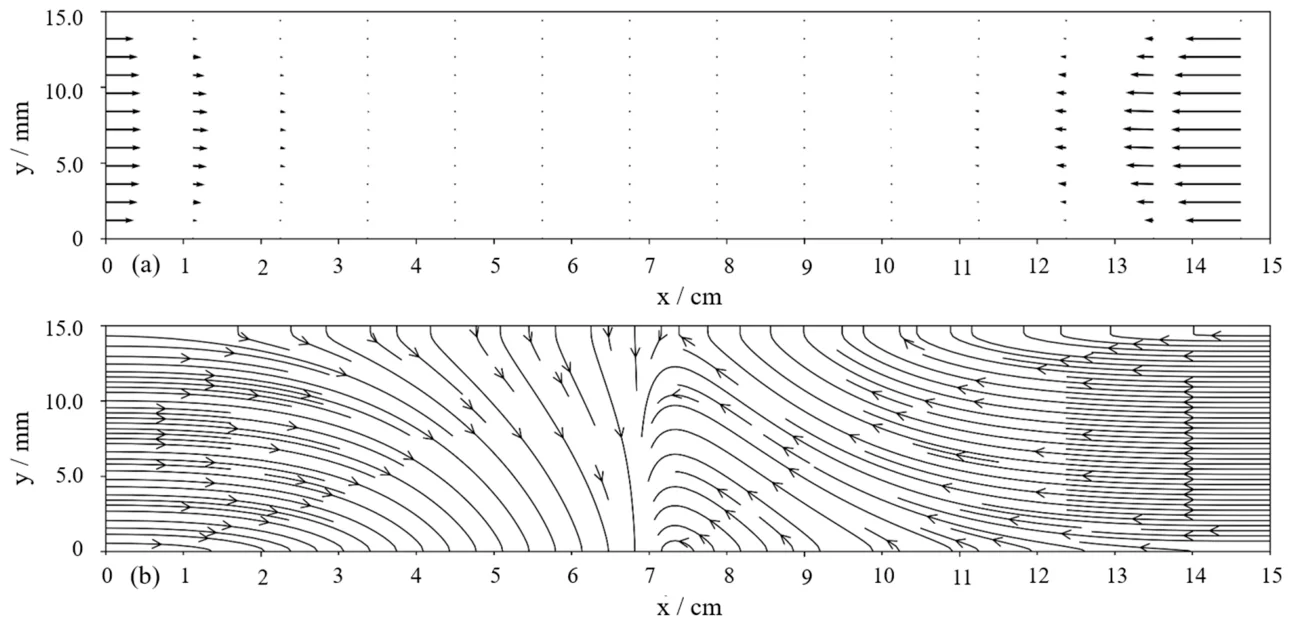
Flow patterns for pure diffusion/advection near the two ends at P_0_ = 1000 Pa (T_s_ = 1123 K, T_c_ = 1773 K, L = 15 and d = 1.5 cm). (**a**) Total velocity discrete plot; (**b**) Total velocity streamline plot.

**Figure 17:**
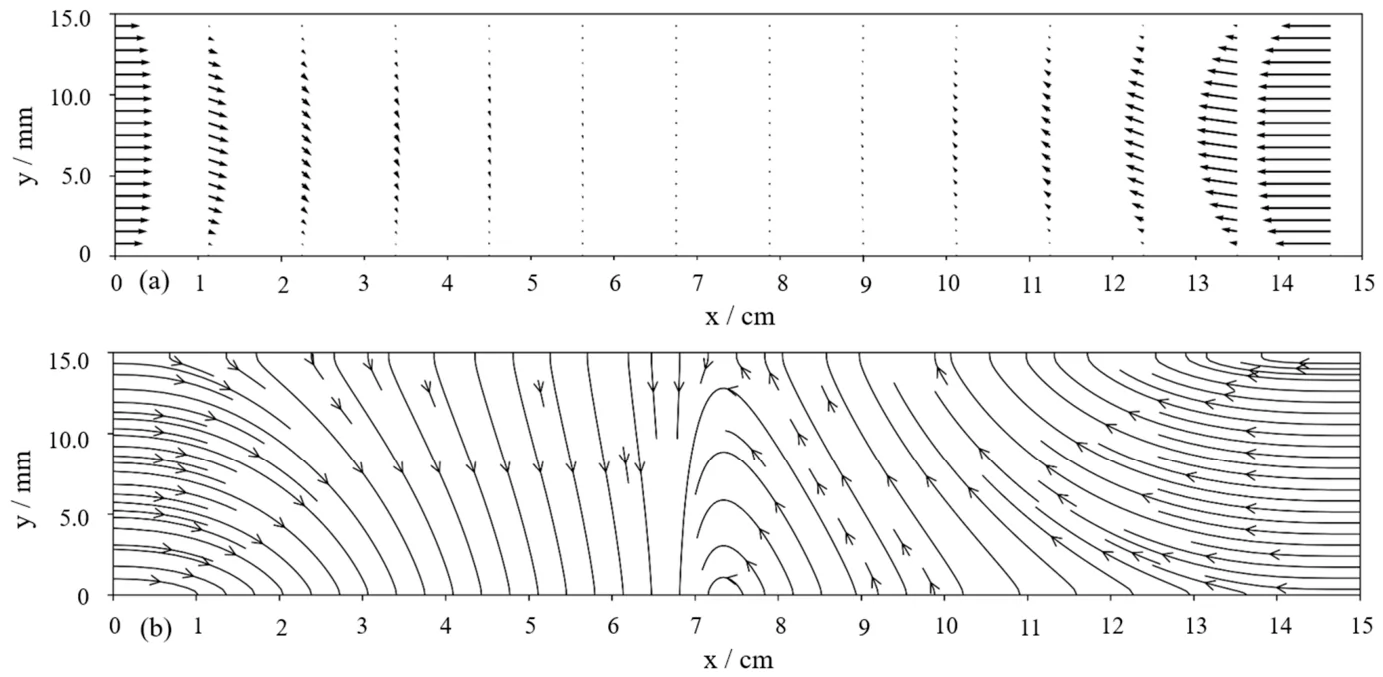
Flow patterns for pure diffusion/advection near the two ends at P_0_ = 3000 Pa (T_s_ = 1123 K, T_c_ = 1773 K, L = 15 and d = 1.5 cm). (**a**) Total velocity discrete plot; (**b**) Total velocity streamline plot.

The evolution of the internal mass transport flow patterns with increasing total system pressure is clearly illustrated in Figure 14, Figure 15, Figure 16 and Figure 17. As the total pressure rises from 300 Pa to 3000 Pa, the flow morphology undergoes a continuous sequence of transitions: horizontal diffusion → slightly curved flow → transitional vortex → fully established recirculation. Figure 14a presents a discrete arrow plot of the total velocity field inside the model. It is observed that arrows near both the source and deposition ends are oriented horizontally toward the opposite end, with longer lengths near the end surfaces and gradually shorter lengths toward the central region, eventually appearing as dots. This is attributed to the higher local velocities driven by the temperature gradients and chemical reactions near the ends; in the central region, mass transport relies mainly on diffusional inertia, resulting in substantially lower velocities (by orders of magnitude) than those near the ends. Thus, the dotted appearance of arrows in the central region does not indicate zero velocity.

Figure 14b shows the total velocity streamlines, which more clearly illustrate the continuity of the actual flow field. Together, Figure 14a,b indicate that at a total system pressure of 300 Pa, the internal flow regime is dominated by pure diffusion and advection, with virtually no buoyancy-driven natural convection. In this regime, the mass transport is entirely governed by horizontal advection driven by the chemical reactions at the source and deposition zones (characteristic of Stefan flow) and by molecular diffusion. Figure 14c further illustrates the respective flow directions of the two gaseous species, VI_2_ and I_2_. The net transport direction of VI_2_ is consistently from the source end to the deposition end (rightward), with transport rate increasing along the path (lengthening arrows). In contrast, the net transport direction of I_2_ is always from the deposition end to the source end (leftward), also with increasing transport rate (lengthening arrows). This clearly demonstrates the cyclic role of I_2_ as the transport agent: it is continuously consumed at the source end and regenerated at the deposition end, then flows back leftward. The rapid consumption of I_2_ near the source end creates a steep partial pressure gradient, accelerating the backflow of I_2_, as reflected by the notably longer blue arrows in the source vicinity in Figure 14c. Similarly, at the deposition end, the partial pressure gradient caused by the continuous deposition of VI_2_ promotes the accelerated migration of VI_2_ toward the deposition surface. Such unidirectional, accelerated migration of the two components gives rise to a “pumping-like” driving effect.

When the total system pressure is increased to 500 Pa (Figure 15), the velocity arrows show a slight deflection in direction in regions slightly away from the two ends, and the streamlines in the total velocity streamline plot begin to exhibit a mild wavy curvature, with a tendency to converge toward the center in the middle part of the model, particularly near the upper and lower walls. This behavior is attributed to the increase in gas density with rising total pressure. According to Equation (17), when Gr approaches its critical value, the buoyancy effect becomes appreciable. Consequently, the internal flow field begins to deviate from the purely horizontal regime, marking the onset of a transition from pure diffusion to a mixed diffusion-convection state.

When the total system pressure is further increased to 1000 Pa (Figure 16), a distinct elliptical vortex circulation begins to emerge in the central region of the model. This indicates that, at 1000 Pa, the Gr has exceeded its critical value (on the order of 10^3^), so that the buoyancy-driven natural convection becomes comparable in strength to the horizontal Stefan flow (horizontal diffusive advection). The two mechanisms exhibit a clear competitive interplay, ultimately resulting in the transitional vortex pattern shown in Figure 16b. It is worth noting that, despite this flow transition, the deflection angle of the transport velocity remains relatively small [Figure 16a], and the net transport of VI_2_ is still dominated by diffusion and advection, with convection playing only a supporting role.

As the total system pressure is further raised to 3000 Pa, the transport velocity undergoes a pronounced directional deflection [Figure 17a], and a closed elliptical vortex circulation is fully established in the central region [Figure 17b], with uniformly distributed streamlines that indicate a strongly convection-dominated regime. At this pressure, the Gr has increased to approximately 10^4^, and the intensity of natural convection significantly exceeds that of the horizontal Stefan flow. As shown in Figure 17b, the streamlines are no longer straight, indicating that the internal mass transport has transitioned from the intermediate state observed at 1000 Pa to a fully convection-dominated mode, where buoyancy-driven circulation becomes the primary mechanism controlling the flow morphology of the transported species.

The gas-phase species flow field and flow pattern transitions obtained in the present simulation are in qualitative agreement with those reported for the ZnS–I_2_ system under comparable CVT conditions [33,34], although direct quantitative comparison is limited owing to differences in the chemical system, operating temperature, and pressure.

### 5.3. Model Assumptions and Limitations

The present two-dimensional rectangular model is a first-order approximation of the actual three-dimensional cylindrical reactor. Under the low-pressure conditions of this study, mass transport is dominated by diffusion and Stefan flow, and this approximation captures the main transport trends. Similar simplifications have been adopted in previous CVT studies, such as the modeling of the ZnS–I_2_ system by Ran Zuo et al. [33,34]. However, in a vertical cylindrical ampoule, gravity-driven natural convection may differ from that in a horizontal enclosure, and in the high-pressure convection-dominated regime, three-dimensional effects may become significant, altering the flow pattern and transport rate. A fully three-dimensional simulation would be necessary in that case.

Also, some errors will be introduced by using the properties of pure I_2_ to represent the VI_2_–I_2_ mixture. Appendix A Table A1 presents the heat capacity, thermal conductivity, and viscosity of the VI_2_–I_2_ mixture as functions of the VI_2_ mole fraction ($x_{{VI}_{2}}$) and mass fraction ($\omega_{{VI}_{2}}$) at representative temperatures of T = 1500 K, together with the relative deviations from the pure I_2_ data used in the simplified model. The properties were calculated using the ideal-gas mixture assumption and the Wilke mixing rule [44]. For VI_2_ mole fractions below 0.6, the relative deviations between the mixture properties and the pure I_2_ approximation are approximately 5–10%. And even when the VI_2_ mole fraction reaches about 0.9, the relative deviations between the mixture and the pure I_2_ approximation for viscosity μ, thermal conductivity k, and constant-pressure heat capacity Cp are only about 8.8%, −8.1%, and −15.2%, respectively. These results indicate that the simplified property model is acceptable for qualitative analysis under the low-pressure conditions of this study, whereas accurate quantitative analysis requires a composition-dependent mixture model.

The contribution of I is neglected in the diffusion and thermophysical property calculations, where only VI_2_ and I_2_ are treated as transporting species. However, the present model does not completely neglect the gas-phase dissociation of I_2_. The equilibrium reaction I_2_ = 2I and the conservation of iodine atoms are included in Equations (5)–(7), and the partial pressure of I is considered in the boundary conditions. At the filament temperature of 1773 K, the thermal dissociation of I_2_ into I is thermodynamically favored and is expected to be significant. Under the low-pressure conditions of this study, the partial pressure of I may not be negligible compared with that of I_2_, and its contribution to the total pressure ∑P and the driving concentration gradient may affect the predicted transport rate. Moreover, I may participate in the heterogeneous reaction V + 2I ⇌ VI_2_ and thus contribute to the vanadium transport flux. A fully coupled model including I transport and composition-dependent mixture properties is necessary for a deeper understanding of the dissociation–transport process.

The assumption of local thermodynamic equilibrium at both the source and the filament is also an idealized treatment. At high deposition rates, the surface reactions may not reach true equilibrium, and the interfacial concentrations and temperature gradients may deviate from the equilibrium values, particularly at the filament where rapid decomposition and deposition of VI_2_ occur.

Despite these simplifications, the simulation results are qualitatively consistent with the experimental observations, suggesting that the main transport trends of VI_2_ are captured. A more comprehensive model that includes I transport, composition-dependent mixture properties, non-equilibrium surface kinetics, and three-dimensional effects where necessary is essential for a deeper understanding of the dissociation–transport process and for improving quantitative accuracy.

## 6. Summary and Conclusions

In this study, the optimal process parameters for the growth of high-purity vanadium crystal bars were determined, the effects of process parameters on the deposition process were clarified, and the coupled diffusion–convection transport mechanism during iodide refining was qualitatively revealed. The main findings are summarized as follows:

(1) Key process parameters and their influence patterns were obtained through optimized experiments. The deposition rate exhibited a bell-shaped dependence on the source temperature, with the optimal source temperature ranging from 1123 to 1173 K and the optimal K value (characterizing the deposition filament temperature) ranging from 40 to 50. At low pressure, the initial iodine charge had only a limited effect, whereas full loading of the feed ensured a uniform atomic flux on the deposition filament surface, effectively preventing tapering or necking of the crystal bar and yielding rods with uniform diameter.

(2) The iodide refining of vanadium metal achieved significant purification. The metallic impurities Fe, Cr, Al, and Si were reduced to 0.025–0.033 wt.%, <0.001 wt.%, <0.001 wt.%, and <0.002 wt.%, respectively, while the interstitial impurities O, C, and N were reduced to <0.01 wt.%, <0.002 wt.%, and <0.002 wt.%, respectively. These results indicate that the process is highly effective in removing both metallic and interstitial impurities.

(3) The mass-transfer mechanism of the vanadium iodide refining process was revealed through two-dimensional numerical simulation using the SIMPLE algorithm. Mass transfer is governed by coupled diffusion and convection. At low pressure (300 Pa), diffusion/advection dominates, whereas with increasing total pressure (up to 3000 Pa), buoyancy-driven circulation gradually develops, and the transition in flow regime corresponds to distinct Grashof number thresholds. The transport rate decreases monotonically with increasing total pressure and with increasing transport distance, but increases with temperature owing to the enhanced VI_2_ partial-pressure difference. The simulation results are qualitatively consistent with the experimental observations, providing a theoretical basis for process optimization.

To further improve the purity and scale-up potential of the process, it is necessary to conduct more in-depth research, including the development of fully coupled models that incorporate gas-phase dissociation, non-equilibrium surface kinetics, and composition-dependent mixture properties, as well as three-dimensional simulations and reactor material optimization.

## Figures and Tables

**Table 1:** The impurity element contents of metal vanadium, wt.%.

Grade	V	Impurity Elements (Not More Than)						
		Fe	Cr	Al	Si	C	N	O
V-4	≥99.0	0.15	0.15	0.08	0.08	-	-	0.10

**Table 2:** Content of impurity elements in raw material iodine, wt.%.

Elements	I	F	Si	C	N
contents	≥99.8	0.005	0.005	0.06	0.04

**Table 3:** The condition parameters selected for the verification experimental.

Parameters	Raw Temperature/K	K Values	Initial Iodine Amount/g	Raw Packing Configuration
selected range	(1) 773 (2) 873 (3) 973 (4) 1023 (5) 1073 (6) 1123 (7) 1173 (8) 1223	(1) 20 (2) 30 (3) 40 (4) 50 (5) 55 (6) 60	(1) 100 (2) 150 (3) 200 (4) 250	(1) bottom filling (2) intermittent filling (3) full loading

**Table 4:** The impurity element contents of raw and crystalline bar, wt.%.

	Impurity Elements (Not More Than)						
	Fe	Cr	Al	Si	C	N	O
Raw	0.15	0.15	0.08	0.08	-	-	0.10
Crystal	0.025–0.033	<0.001	<0.001	<0.002	<0.002	<0.002	<0.01

**Table 5:** Variables and constants corresponding to Equation (8) in different cases.

	φ	Γ	S
Mass conservation	1	0	0
x-momentum conservation	u	μ	− ∂p/∂x
y-momentum conservation	v	μ	− ∂p/∂y − ρgy
Energy conservation	T	k/c_p_	0
Mass component conservation	ω	ρD_ij_	0

## Data Availability

The original contributions presented in this study are included in the article. Further inquiries can be directed to the corresponding author.

## Appendix A

**Table A1:** Thermophysical properties of the VI_2_-I_2_ mixture and their relative deviations from the simplified model at T = 1500K.

$x_{{VI}_{2}}$	$\omega_{{VI}_{2}}$	μ_mix_/kg · m^−1^ · s^−1^	Relative Deviation of μ	k_mix_/W · m^−1^ · K^−1^	Relative Deviation of k	C_p,mix_/J · kg^−1^ · K^−1^	Relative Deviation of C_p_
0	0	5.64 × 10^−5^	0.0%	6.92 × 10^−3^	0.0%	114.5	0.0%
0.1	0.12	5.69 × 10^−5^	1.0%	6.85 × 10^−3^	−1.0%	112.27	−1.9%
0.2	0.23	5.75 × 10^−5^	2.1%	6.78 × 10^−3^	−1.9%	110.11	−3.8%
0.3	0.34	5.81 × 10^−5^	3.1%	6.72 × 10^−3^	−2.9%	108.04	−5.6%
0.4	0.44	5.86 × 10^−5^	4.0%	6.66 × 10^−3^	−3.8%	106.05	−7.4%
0.5	0.55	5.92 × 10^−5^	5.0%	6.59 × 10^−3^	−4.7%	104.13	−9.1%
0.6	0.64	5.97 × 10^−5^	6.0%	6.53 × 10^−3^	−5.5%	102.28	−10.7%
0.7	0.74	6.03 × 10^−5^	6.9%	6.48 × 10^−3^	−6.4%	100.49	−12.2%
0.8	0.83	6.08 × 10^−5^	7.9%	6.42 × 10^−3^	−7.2%	98.76	−13.7%
0.9	0.91	6.13 × 10^−5^	8.8%	6.36 × 10^−3^	−8.1%	97.09	−15.2%
1	1	6.18 × 10^−5^	9.7%	6.30 × 10^−3^	−8.9%	95.49	−16.6%
